# Metal‐Organic Fragments with Adhesive Excipient and Their Utilization to Stabilize Multimetallic Electrocatalysts for High Activity and Robust Durability in Oxygen Evolution Reaction

**DOI:** 10.1002/advs.202100044

**Published:** 2021-03-24

**Authors:** Won Ho Choi, Keon‐Han Kim, Heebin Lee, Jae Won Choi, Dong Gyu Park, Gi Hwan Kim, Kyung Min Choi, Jeung Ku Kang

**Affiliations:** ^1^ Department of Materials Science and Engineering Korea Advanced Institute of Science and Technology (KAIST) 291 Daehak‐ro, Yuseong‐gu Daejeon 34141 Republic of Korea; ^2^ Department of Chemical and Biological Engineering Sookmyung Women's University Cheongpa‐ro 47‐gil 100, Yongsan‐gu Seoul 04310 Republic of Korea

**Keywords:** adhesive excipient, high activity and robust durability, metal‐organic fragment, multimetallic electrocatalysts, oxygen evolution reaction

## Abstract

Multimetallic electrocatalysts have shown great potential to improve electrocatalytic performance, but their deteriorations in activity and durability are yet to be overcome. Here, metal‐organic fragments with adhesive excipient to realize high activity with good durability in oxygen evolution reaction (OER) are developed. First, a leaf‐like zeolitic–imidazolate framework (ZIF‐L) is synthesized. Then, ionized species in hydrogen plasma attack preferentially the organic linkers of ZIF‐L to derive cobalt–imidazole fragments (CIFs) as adhesive excipient, while they are designed to retain the coordinated cobalt nodes. Moreover, the vacant coordination sites at cobalt nodes and the unbound nitrogen at organic linkers induce high porosity and conductivity. The CIFs serve to stably impregnate trimetallic FeNiMo electrocatalysts (CIF:FeNiMo), and CIF:FeNiMo containing Fe contents of 22% and hexavalent Mo contents show to enable high activity with low overpotentials (203 mV at 10 mA cm^−2^ and 238 mV at 100 mA cm^−2^) in OER. The near O K‐edge extended X‐ray absorption fine structure proves further that high activity for OER originates from the partially filled e_g_ orbitals. Additionally, CIF:FeNiMo exhibit good durability, as demonstrated by high activity retention during at least 45 days in OER.

## Graphical abstract

A strategy is developed to produce metal‐organic fragments allowing to stabilize multimetallic electrocatalysts in which a leaf‐like zeolitic–imidazolate framework is transformed into Co–imidazole fragments (CIFs) with unsaturated orbitals and dangling bonds as adhesive excipient. Fe, Ni, and Mo elements impregnated within CIFs, referred as CIF:FeNiMo catalysts, demonstrate high activity with low overpotentials (203 mV at 10 mA cm^−2^ and 238 mV at 100 mA cm^−2^) and robust durability with high activity retention (during at least 45 days) in oxygen evolution reaction.

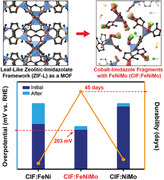

## 1 Introduction

Significant attention has been directed toward the development of electrocatalysts for the production of value‐added chemical fuels such as hydrogen,^[^ ^1^, ^2^ ^]^ hydrocarbons,^[^ ^3^, ^4^ ^]^ and ammonia^[^ ^5^ ^]^ from H_2_O, CO_2_, and NO_2_, respectively. Among the promising electrocatalyst candidates, multimetallic electrocatalysts have shown great potential to realize high efficiency because the incorporation of multiple metal components allows the control of activity.^[^ ^6^, ^7^, ^8^, ^9^ ^]^ However, previous studies^[^ ^10^, ^11^ ^]^ have shown that multimetallic electrocatalysts suffer from the fast leaching of active metals, resulting in phase segregation. In turn, this leads to deteriorations in activity and durability due to the altered surface composition and low electrical conductivity. Therefore, adhesive excipients have attracted considerable attention for stabilizing multimetallic electrocatalysts to overcome the above challenges. The use of adhesive recipients with specific pore architectures can not only increase the number of exposed electrocatalytic sites, but also prevent phase segregation.^[^ ^12^, ^13^, ^14^ ^]^ The previous results suggest that adhesive excipients must have high chemical and physical affinity to induce strong interactions with the multimetallic electrocatalysts. To date, the creation of abundant high‐affinity sites for anchoring electrocatalysts in the porous supporting media remain challenging because conventional materials such as carbon black have affinity only at specific positions, limiting the number of high‐affinity sites. Although affinitive sites can be created by chemical modification, they are easily degraded via carbon‐corrosion and aggregation. Consequently, porous material with high affinity for anchoring electrocatalysts is critical to achieve high electrocatalytic performances without compromising activity.

We hypothesize that metal‐organic fragments with abundant unsaturated orbitals and dangling bonds can serve as porous support materials with high affinity for electrocatalysts that form strong bonds with multimetallic catalysts. Moreover, we expect the unbound orbitals of the metal nodes to enhance electron transport, thereby facilitating electrochemical catalysis by providing delocalized electrons and vacant orbitals.^[^ ^15^, ^16^ ^]^ In principle, adhesive excipient could be created by reorganizing metal‐organic frameworks (MOFs) into smaller pieces. Accordingly, in this work, a pathway for generating metal‐organic fragments as adhesive excipient rom MOF was developed. First, a leaf‐like zeolitic–imidazolate framework (ZIF‐L) composed of cobalt (Co) and 2‐methylimidazole (2‐mim) is synthesized and then transformed into Co–imidazole fragments (CIFs) with unsaturated orbitals and dangling bonds as adhesive excipient. Meanwhile, it was controlled to retain the coordinated metal nodes of ZIF‐L. The vacant orbitals of the metal nodes and the unbonded electrons of the organic linkers resulted in CIFs with high conductivity. Subsequently, CIFs were combined with multiple metal components to form CIF:electrocatalyst complexes. These complexes exhibited high activity and good durability in oxygen evolution reaction (OER).

## 2 Result and DIscussion

**Figure 1:**
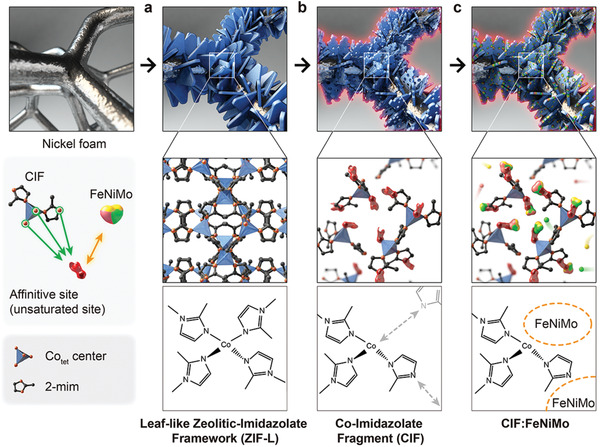
Schematic representation of the overall synthesis steps for the production of a) ZIF‐L, b) CIF, and c) CIF:FeNiMo from ZIF‐L with tetrahedrally coordinated Co center (Co_tet_) and 2‐methylimidazole (2‐min) organic linker.

Co‐based ZIF‐L structures were synthesized directly on Ni foam (NF) by a seeding growth method where a seed layer was coated on the NF surface and then ZIF‐L structures were grown in one direction. ZIF‐L is a MOF structure assembled by the 2D layers formed via hydrogen bonding induced by unsaturated 2‐mim.^[^ ^17^ ^]^ ZIF‐L has rich Co–imidazolate units (**Figure** **1**a) and Figure 1b shows an illustrative synthesis step for the production of CIFs from ZIF‐L. Each CIF, which consists of tetrahedral Co and partially coordinated 2‐mim, possesses highly affinitive sites derived from the unsaturated orbitals and dangling bonds. In addition, the synthesis process induces porous spaces between CIFs that can be easily accessible by guest molecules. When using the highly porous CIFs with adhesive excipient, inorganic metal ions such as iron (Fe), Ni, and molybdenum (Mo) can combine with CIFs to form multimetallic electrocatalysts such as CIF:FeNiMo (Figure 1c).

**Figure 2:**
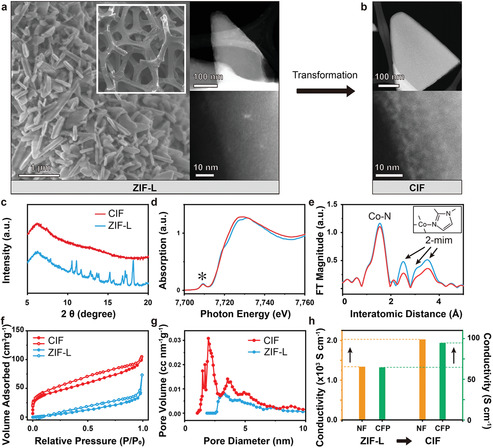
Structural characteristics of Co‐based ZIF‐L and CIF. SEM and HAADF‐STEM images of a) ZIF‐L and b) CIF. c) XRD patterns, d) normalized Co K‐edge XANES spectra, e) radial distribution functions obtained by the Fourier transformation of the *k* ^3^‐weighted Co EXAFS spectra, f) nitrogen physisorption isotherms at 77 K, g) pore size distribution curves calculated by the DFT method, and h) electrical conductivity comparison plot for ZIF‐L and CIF.

The scanning electron microscopy (SEM) image in **Figure** **2**a shows that the fully covered ZIF‐L planar arrays were grown directly on a porous NF. Also, the high‐angle annular dark‐field scanning transmission electron microscopy (HAADF‐STEM) and high‐resolution STEM (HR‐STEM) images (Figure S1, Supporting Information) show the dense triangular planar morphology of ZIF‐L. H_2_ plasma, which gains high energy through repeated collisions with electrons and hydrogen atoms, was employed to tear ZIF‐L into CIFs. Through low‐temperature (<200 °C) bombardments using H_2_ plasma, we were able to achieve the sectional deformation of a ZIF‐L by preventing unpredictable thermal structural destruction. After H_2_ plasma treatment, the dense structure of ZIF‐L was converted into a partially split structure by maintaining the initial 2D structure (Figure 2b; Figures S2–S5, Supporting Information). The X‐ray diffraction (XRD) and X‐ray absorption fine structure (XAFS) spectroscopy were conducted to probe the orders of ZIF‐L and CIF structures. The XRD patterns, which can be used to confirm the long‐range order (crystallinity), show that the characteristic peaks in the spectrum of ZIF‐L are absent in the spectrum of CIF (Figure 2c). In contrast, the pre‐edge peak corresponding to the 1s–3d electric dipole‐forbidden transition in the Co K‐edge X‐ray absorption near‐edge structure (XANES) spectrum of CIF, which originates from the non‐centrosymmetric tetrahedral local structure of ZIF‐L, was preserved after H_2_ plasma bombardment (asterisk in Figure 2d). These conflicting results reveal that high‐energy H_2_ plasma destroyed the long‐range order of ZIF‐L but had only a slight effect on the tetrahedral units. C 1s and N 1s XPS spectra clarify how the H_2_ plasma affected 2‐mim within CIF. Figure S6 (Supporting Information) shows the new shoulder peaks in the left and right sides of the prominent peak at 284.8 eV originated from sp^2^ C=N bond, indicating the disorders in carbon structures. The more pronounced difference was also observed in N 1s XPS spectra (Figure S7, Supporting Information). The spectrum of ZIF‐L could be deconvoluted into A and B peaks centered at 400.4 and 399.3 eV, in which A peak represents the hydrogen‐bonded nitrogen of 2‐mim and B peak illustrates the tetrahedrally coordinated nitrogen with Co center. Interestingly, in the spectrum of CIF, A peak disappears and C peak appears at 398.3 eV corresponding to the unbounded pyridinic nitrogen. The IR spectra (Figure S8, Supporting Information) shows further that the shoulder peak in C 1s XPS spectrum and C peak in N 1s XPS spectrum were caused by the disordered structure of 2‐mim. The peaks indicated by the arrows are assigned to the C—N stretching mode (1100 and 1174 cm^–1^), C=N stretching mode (1566 cm^–1^), and C—H stretching mode (2924 cm^–1^). All the peaks were weakened and even disappeared, indicating that the original structure was slightly modified with the disorders. Although H_2_ plasma treatment was operated at low temperature, the local sites of ZIF‐L were partially disordered due to the high energetic plasma bombardment, but not fully destroyed. Furthermore, the IR spectra corresponding to the fingerprint area (Figure S9, Supporting Information) shows that ZIF‐L shows the N—Co—N stretching mode at 424 cm^–1^,^[^ ^18^ ^]^ whereas that of CIF shows the blue shifting N—Co—N stretching mode. The blue shift means the higher electron density of Co of CIF than that of ZIF‐L, indicating electron‐rich environment on Co—N bonds in CIFs compared to that on N—Co—N bonds in ZIF‐L. The Co 2p XPS spectra also support the increase in delocalized electrons; the observed shift in the XPS spectrum toward higher energy indicates a reduction in valence state resulting from more filled electrons in divalent Co (Figure S10, Supporting Information). This change results in the greater electron density near a central Co atom, the enhanced shielding effect, and the lower binding energy of a core electron, as demonstrated by the red‐shifted absorption edge.^[^ ^19^ ^]^ The white line in the XANES spectrum of CIF was enhanced compared to in the spectrum of ZIF‐L, indicating a change in coordination environment. As shown in Figure 2e, the coordination environments of ZIF‐L and CIF were investigated by the K‐edge extended X‐ray absorption fine structure (EXAFS) spectroscopy. The main peak in both spectra at 1.65 Å corresponds to the Co—N bond and the other peaks corresponding to 2‐mim are slightly attenuated in the spectrum of CIF compared to in the spectrum of ZIF‐L. This change confirms that the coordinated 2‐mim molecules in ZIF‐L were partially damaged or detached, implying that the Co centers within the CIFs were more exposed than those in ZIF‐L. Moreover, the retention of the 2‐mim peaks after plasma treatment demonstrates that ZIF‐L was not converted into another phase such as CoO or Co_3_O_4_. In the thermogravimetric analysis (TGA) curves, the weight loss at 230 °C is only observed for ZIF‐L; this weight loss can be attributed to the loss of monodentate 2‐mim (Figure S11, Supporting Information). The unsaturated 2‐mim molecules along the c‐axis are more susceptible to structural deformation by H_2_ plasma because monodentate linkers are more thermodynamically unstable than bidentate linkers.^[^ ^20^ ^]^ In the TGA curve of CIF, no weight loss is observed until 490 °C, supporting the absence of unsaturated 2‐mim (Figure S7, Supporting Information). This structural modification indicates that CIFs were separated from each other without hydrogen bonds; the high affinity resulted from the abundance of unsaturated orbitals and dangling bonds. The N_2_ adsorption/desorption isotherms clearly show the change in porosity after H_2_ plasma treatment (Figure 2f). At low relative pressure (*P*/*P*_0_ ≤ 0.1), ZIF‐L showed negligible adsorption, while CIF exhibited enhanced absorption indicative of microporosity. A low‐pressure hysteresis, which was commonly observed in the isotherm of MOFs, indicates the presence of mesopores. The hysteresis occurs by pore condensation and evaporation so that the position of hysteresis appears at low pressure due to the pore blockage occurring in narrow mesopores.^[^ ^21^ ^]^ The mesopores were induced by the detached or damaged 2‐mim, limiting N_2_ kinetics, thereby resulting in low pressure hysteresis. The wide pore distribution of CIF resulted in a type II isotherm showing the coexistence of micropores and mesopores, in contrast to the type III isotherm of ZIF‐L. Comparing the pore size distributions of ZIF‐L and CIF indicates an increase in pore volume in both the micropore and mesopore regions after H_2_ plasma treatment (Figure 2g). The *t*‐plot method was used to distinguish porosity (Table S1, Supporting Information). H_2_ plasma treatment increased the micropore surface area from 0.024 to 56.5 m^2^ g^−1^ and the specific surface area from 63.2 to 159.5 m^2^ g^−1^; thus, micropores and mesopores were formed simultaneously during H_2_ plasma treatment. Interestingly, simple thermal treatment did not induce the same changes in properties, suggesting that the unique characteristics of H_2_ plasma treatment (e.g., ion bombardment and the erosion effect) resulted in new porosity and high‐affinity sites (Figure S12, Supporting Information). When ZIF‐L was exposed to H_2_ plasma, the H_2_ plasma diffused toward the layered structure of ZIF‐L. The high‐energy H_2_ plasma then broke the hydrogen bonds and triggered the formation of CIFs. To determine whether the H_2_ plasma affected the electrical conductivity, four‐point probe measurements were conducted (Figure 2h). The electrical conductivities of ZIF‐L loaded on NF and carbon fiber paper were 1.337 × 10^3^ and 64.6 S cm^−1^ at 298 K, respectively; after H_2_ plasma treatment, the corresponding values for CIF were dramatically increased to 2.025 × 10^3^ and 94.7 S cm^−1^, respectively. Although the electrical conductivities of ZIF‐L and CIF differed greatly based on the type of support, the increases in conductivity upon plasma treatment were significant (151.4% and 146.5% for NF and carbon fiber paper supports, respectively). The improved conductivity was caused by the electron‐rich environment in CIF containing pyridinic nitrogen and vacant coordination sites. These results demonstrate that H_2_ plasma effectively transformed ZIF‐L into highly affinitive, porous, and conductive CIFs.

**Figure 3:**
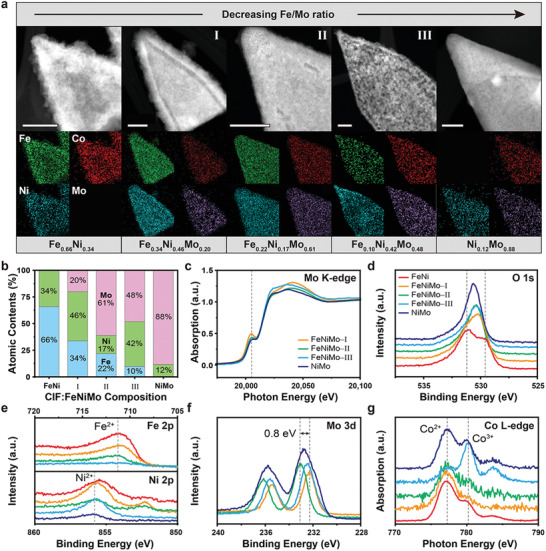
Structural characteristics with different Fe/Mo ratios. a) HAADF‐STEM images (scale bars are 50 nm) and EDX images, b) comparison plot of atomic contents, c) O 1s XPS spectra, d) O 1s XPS spectra, e) Fe 2p and Ni 2P XPS spectra, f) Mo 3d XPS spectra, and g) normalized Co L‐edge NEXAFS spectra of FeNi, FeNiMo‐I, FeNiMo‐II, FeNiMo‐III, and NiMo.

CIFs were employed as host structures with adhesive excipient by forming CIF:FeNiMo. CIFs were immersed in precursor solutions with different Fe/Mo ratios, in which the Fe content was fixed and the Mo content was altered. Ni ion was spontaneously supplied via the corrosion of NF. It is well known that metallic Ni is dissolved as chloride ions react due to the strong penetrating ability of chloride ions.^[^ ^22^ ^]^ Each composition (FeNi, FeNiMo‐I, FeNiMo‐II, FeNiMo‐III, and NiMo) was easily obtained using the simple wet‐chemical synthetic process. The TEM and energy‐dispersive X‐ray spectroscopy (EDX) analyses show that the three metal components were homogeneously dispersed throughout the structure (**Figure** **3**a; Figures S13–S17, Supporting Information). When the Fe/Mo ratio was high (or only Fe ions were used), thin, sheet‐like layers were deposited on the CIF surface because the plentiful Fe ions quickly formed hydroxides with Ni before the formation of FeNiMo. These sheet‐like layers began to disappear as the Fe/Mo ratio decreased, and the sheets were completely absent in FeNiMo‐II. Further decreasing the Fe/Mo ratio (beyond FeNiMo‐II) resulted in the formation of large pores in FeNiMo‐III and the deposition of dense layers on the CIF surface in the case of NiMo. Together, the results indicate that controlling the Fe/Mo ratio allowed FeNiMo to be homogenously impregnated within the CIFs without by‐products or additional organic materials, especially in the case of FeNiMo‐II (see the C 1s XPS spectra in Figure S18 in the Supporting Information). The results imply that the Fe/Mo ratio is a crucial factor in the appropriate incorporation of FeNiMo in CIFs. The XRD patterns and HR‐TEM images indicate that all compositions have the amorphous characteristics (Figures S19–S24, Supporting Information), which have the structural advantages such as fast charge transfer pathways^[^ ^23^, ^24^ ^]^ and abundant defect sites^[^ ^25^ ^]^ to design high‐performance electrocatalysts. Regarding the effects of the Fe/Mo ratio on composition, three notable trends were observed (Figure 3b). First, the Fe content tended to decrease gradually with decreasing Fe/Mo ratio. Second, the changes in Ni and Mo contents were inversely proportional to each other. Lastly, FeNiMo‐II showed a significantly increased Mo content, while Fe and Ni continued to decrease compared to Mo. The above three trends suggest that Mo was the most influential element in the FeNiMo composition. Mo easily coordinates with oxygen in the form of MoO*_x_*,^[^ ^26^ ^]^ which is known to decorate NiFe alloy nanosheets^[^ ^27^ ^]^ and diffuse amorphous NiFe.^[^ ^28^ ^]^ The Mo K‐edge XANES (Figure 3c) and EXAFS (Figure S25, Supporting Information) spectra confirmed the existence of the hexavalent oxidation state and Mo—O bonds. In the XANES spectra, the intense pre‐edge peak at 20 005 eV implies that hexavalent Mo promoted the 1s–4d quadruple transition.^[^ ^29^ ^]^ The *k* ^3^‐weighted normalized EXAFS spectra supported the presence of Mo—O bonds within octahedrally coordinated mononuclear MoO_6_ without additional shells. Therefore, mononuclear MoO_6_ was the dominant compound deciding the morphology and composition and played the most crucial role in forming FeNiMo. In the O 1s XPS spectrum of FeNi (Figure 3c), the two peaks observed at 531.2 and 529.8 eV correspond to protonated and unprotonated oxygen, respectively. The presence of these separate oxygen peaks indicates the existence of independent O‐containing phases.^[^ ^11^ ^]^ Such phase segregation is consistent with the Fe‐rich islands that occur when a large amount of Fe ions are incorporated into an Ni‐based lattice.^[^ ^30^ ^]^ As the MoO_6_ was further employed to FeNiMo, the two peaks merged into a single peak, indicating that the oxo‐group of MoO_6_ served as a structural frame for FeNiMo without Fe‐ or Ni‐based by‐products. As more MoO_6_ participated, the O 1s XPS spectrum shifted toward higher energy; this trend is also seen in the Fe 2p and Ni 2p XPS spectra (Figure 3d). Interestingly, the Mo 3d XPS spectrum of FeNiMo‐II only located in 233.1 eV as hexavalent Mo, and other spectra of FeNiMo‐I and III are lower shifted as 0.8 eV (Figure 3e). These contradictory shifts in the spectra of FeNiMo‐I and FeNiMo‐III imply that MoO_6_ attracted the electrons of divalent Fe and Ni to compensate for the deficient electron density. As the Mo content in FeNiMo‐II increased obviously with respect to Fe and Ni, the oxidation state of Mo remained hexavalent, even after the extraction of electrons from Fe and Ni. Maintaining the hexavalent state is critical to OER activation because hexavalent Mo effectively optimizes the adsorption energy for intermediates^[^ ^30^ ^]^ by redistributing electrons within FeNiMo through oxo‐groups. In addition, the introduction of a high‐valent third metal into the tri‐metallic electrocatalyst is a well‐known practical approach to improving stability due to its versatile functions such as holding other metals and tuning electronic structure.^[^ ^31^, ^32^ ^]^ The Co L_2,3_‐edge near‐edge X‐ray absorption fine structure (NEXAFS) spectrum of FeNiMo‐III shows a new transition at 780.1 eV corresponding to Co^3+^ (Figure 3g).^[^ ^18^ ^]^ When the amount of Fe was not sufficient to compensate for the electron density of hexavalent Mo, the MoO_6_ invaded the Co center in CIFs. As a result, Co^3+^ and larger pores appeared in FeNiMo‐III. When only Mo was used, NiMo accounted for 88% of Mo, and no Co^3+^ transition was observed. This indicates that MoO_6_ existed as a thin layer with mixed oxidation states. In order to confirm the role of CIF, we have also synthesized FeNiMo having no CIF. The SEM image (Figure S26, Supporting Information) shows the etched NF surface instead of the formation of FeNiMo by chloride ions. This indicates that the high affinitive sites of CIF strongly pull Fe, Ni, and MoO_6_ to form tri‐metallic FeNiMo, while preventing the NF corrosion.

**Figure 4:**
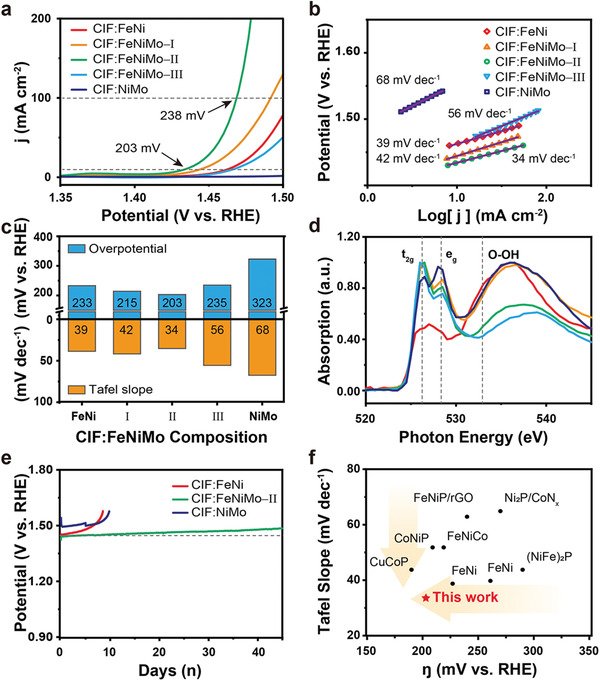
Electrochemical characteristics. a) OER polarization curves at 1 mV s^−1^, b) Tafel plots inferred from (a), c) overpotential and Tafel slopes, and d) normalized O K‐edge NEXAFS spectra of CIF:FeNi, CIF:FeNiMo‐I, CIF:FeNiMo‐II, CIF:FeNiMo‐III, and CIF:NiMo. e) Prolonged chronopotentiometry curves of CIF:FeNi, CIF: FeNiMo‐II, and CIF:NiMo. f) Comparison of the electrocatalytic performance obtained in this work with those of MOF‐derived materials in the literature.

**Figure** **4**a shows the polarization curves of CIF:FeNi, CIF:FeNiMo‐I, CIF:FeNiMo‐II, CIF:FeNiMo‐III, and CIF:NiMo. Compared to NiMo, which does not have any Fe component, FeNiMo‐I, FeNiMo‐II, FeNiMo‐III, and FeNi promoted the OER activity. Fe is regarded as an active site in multi‐metallic systems, and the OER performance of FeNiMo is best when the Fe content is in the range of 10%–30%.^[^ ^11^, ^33^ ^]^ Our results are consistent with these past studies; FeNiMo‐II, which had an Fe content of 22%, exhibited the lowest overpotential (203 mV at 10 mA cm^−2^ and 238 mV at 100 mA cm^−2^) among the studied electrocatalysts (Figure 4a). In contrast to the activity trends, the Ni component did not affect the increase in OER activity, consistent with recent reports.^[^ ^30^, ^34^ ^]^ Furthermore, the Tafel slope for FeNiMo‐II was 34 mV dec^−1^, in agreement with the theoretical value of 30 mV dec^−1^. This suggests that OER involves a chemical oxide‐based pathway with a recombination step (Figure 4b).^[^ ^35^ ^]^ As the composition changed, the Tafel slope changed to approximately 40 or 60 mV dec^−1^ and even to ≈70 mV dec^−1^ for NiMo. The Tafel slopes of 40 mV dec^−1^ for FeNi and FeNiMo‐I can be attributed to the low OH_ads_ coverage due to hindrance by the sheet‐like deposits. The Tafel slope of over 60 mV dec^−1^ for FeNiMo‐III is typical for Co‐based materials and implies that Co^3+^ in FeNiMo‐III acted as an active site with one electron/proton step.^[^ ^36^ ^]^ These diverse Tafel slopes indicate that the rate‐determining step varied with electrocatalyst composition, and the smallest Tafel slope resulted in the fastest kinetics in CIF:FeNiMo‐II (Figure 4c). To obtain efficient OER, many strategies based on the modulation of d electrons, average bond length, ionization energy, and so forth have been investigated.^[^ ^37^, ^38^ ^]^ Recently, the filling of the e_g_ orbital of the 3d active metal has emerged as a promising strategy based on both experimental and theoretical findings. The e_g_ electrons of active transition metals promote electron transfer because of their stronger overlap with oxygen‐related adsorbates compared to the t_2g_ orbital.^[^ ^39^ ^]^ Thus, O K‐edge NEXAFS spectroscopy is suitable in this case because localized e_g_ electrons direct toward the orbitals of oxygen.^[^ ^40^ ^]^ The O K‐edge NEXAFS spectrum shows a doublet corresponding to the transition of the core electron into the lowest unoccupied t_2g_ and e_g_ orbitals in the MoO_6_ octahedral symmetry (Figure 4d).^[^ ^41^ ^]^ The peak corresponding to the transition to the e_g_ orbital was diminished, consistent with the decreasing trend in Fe content. This shows that e_g_ orbital filling depends on the Fe contents, suggesting that CIF:FeNiMo‐II containing an Fe content of 22% effectively leads to optimize the adsorption and desorption energies of the intermediates for high OER activity. Based on these findings, the optimal t_2g_/e_g_ ratio can be used as a criterion for evaluating OER characteristics. In this study, the t_2g_/e_g_ ratio of FeNiMo‐II was 0.809, while those of FeNiMo‐I, FeNiMo‐III, and NiMo were 0.865, 0.756, and 1.088, respectively. The value of 0.809 for FeNiMo‐II confirms that the best OER performance was obtained when the e_g_ orbital was partially filled rather than completely empty since the intermediates overlap with partially filled e_g_ orbitals.^[^ ^42^, ^43^ ^]^ The subsequent transition in the high‐photon‐energy region over t_2g_ and e_g_ indicates that the antibonding combination of direct oxygen–oxygen interaction was dominant in FeNi, FeNiMo‐I, and NiMo. The enhanced oxygen‐oxygen interaction imply that sheet‐like products were formed as –OOH phase based. Durability is another important factor when evaluating multi‐metallic electrocatalysts. In a prolonged chronopotentiometry test, CIF:FeNiMo‐II exhibited excellent durability at a constant current density of 10 mA cm^−2^ for over 45 days (Figure 4e). After 45 days, CIF:FeNiMo‐II showed a slightly increased overpotential of 40 mV due to oxygen saturation and low KOH concentration. The durability of CIF:FeNiMo‐II was much better than those of CIF:FeNi and CIF:NiMo, which showed rapid increases in overpotential after 10 days. The good durability of CIF:FeNiMo‐II is derived from the synergetic effect of the highly affinitive CIFs, the hexavalent Mo, and partially filled e_g_ orbital, which facilitated fast kinetics.^[^ ^33^ ^]^ The chart in Figure 4f compares the overpotentials and Tafel slopes of various MOF‐derived electrocatalysts.^[^ ^44^, ^45^, ^46^, ^47^, ^48^, ^49^, ^50^, ^51^ ^]^ Although some MOF‐derived electrocatalysts present good activity or kinetics, the overpotential and Tafel slope of CIF:FeNiMo‐II are significantly improved with respect to those of previously reported MOF‐based electrocatalysts. Table S2 (Supporting Information) shows that CIF:FeNiMo‐II leads to excellent activity, as demonstrated by the lowest overpotential for OER among previously reported MOF‐based electrocatalysts. In addition, the Tafel slope of less than 40 mV dec^–1^ supports that the rapid OER occurs as the active sites are closer to each another,^[^ ^52^, ^53^ ^]^ suggesting that the close Fe sites of CIF:FeNiMo‐II provide abundant adsorption sites for aqueous hydroxide. The presence of Mo made it possible to maintain the stable single‐phase FeNiMo during OER using electron redistribution ability by the hexavalent states. Additionally, the tuned electronic structure is explained by the partially filled e_g_ orbitals depending on the Fe contents of CIF:FeNiMo and leads to the optimized adsorption strength of the intermediates. As a result of abundant Fe sites, hexavalent Mo, and partially filled e_g_ orbitals, CIF:FeNiMo‐II showed high performances in overpotential, Tafel slope, and durability simultaneously.

## 3 Conclusion

In summary, we developed a strategy to produce metal‐organic fragments for stabilizing trimetallic FeNiMo via H_2_ plasma. The strategy successfully tore ZIF‐L into porous and conductive CIFs with high‐affinity sites for FeNiMo while retaining the tetrahedrally coordinated Co nodes. Subsequently, tri‐metallic FeNiMo was impregnated within the CIFs. Finally, when used for OER, CIF:FeNiMo‐II exhibited high activity and fast kinetics with the low overpotentials of 203 mV at 10 mA cm^−2^ and 238 mV at 100 mA cm^−2^. The degree of Fe contents and mononuclear MoO_6_ within CIF:FeNiMo‐II play as key factors for high activity and good durability, respectively. The O K‐edge NEXAFS spectroscopy analysis proved that the partially filled e_g_ orbitals of CIF:FeNiMo‐II governed the balance between the adsorption and desorption energies of the intermediates during OER. Moreover, CIF:FeNiMo‐II showed good durability, as demonstrated by at least 45 days without the loss of activity. Consequently, this study paves a route to build highly affinitive sites on the porous material platform for tri‐metallic electrocatalysts, and the platform impregnating electrocatalyst complexes show high activity and good durability.

## 4 Experimental Section

##### Materials

Cobalt(II) nitrate hexahydrate (Co(NO_3_)_2_·6H_2_O, 98+%), 2‐methyleimidazole (2‐mim, 99%), Iron(III) chloride (FeCl_3_, 97+%), Molybdenum(V) chloride (MoCl_5_, 99.99%), Pure ethanol were purchased from Sigma‐Aldrich. Nickel foam (1.6 mm thickness) was purchased from MTI Korea. Deionized water (d.i. water) was obtained from a purifying system using UV treatment. All chemicals were used without further purification.

##### Preparation of Co‐Based ZIF‐L

First, precursor solutions of 3 × 10^−3^ m Co(NO_3_)_2_ and 800 × 10^−3^ m 2‐mim in d.i. water were prepared. Two solutions were poured in petri dish (10 cm × 10 cm) at room temperature. The mixture solution immediately turned purple. Then, NF (5 cm × 5 cm) was soaked in the mixed solution and was kept for 1 h at room temperature. Subsequently, the nickel foam was rinsed with ethanol three times and was dried overnight in vacuum oven at 60 °C.

##### Synthesis of CIF

First, two glass pieces (0.5 cm × 1 cm) were placed on silicon wafer (10 cm × 6 cm), then Co‐based ZIF‐L on NF (5 cm × 2 cm) was placed on the top of the glass pieces. After that, two other glass pieces were stacked on top of Co‐based ZIF‐L on NF, and covered with another silicon wafer (7 cm × 3 cm). The piled substrate was placed in the chamber of microwave plasma enhanced chemical vapor deposition (MPE‐CVD) equipment. Then, H_2_ plasma was generated above Co‐based ZIF‐L on nickel foam at a microwave power of 500 W with H_2_ (99.999%, 89 sccm) for 13 min. After exposure to H_2_ plasma, the color of Co‐based ZIF‐L was changed from purple to blue. After cooled down to room temperature, the CIFs were pull out from the chamber of MPE‐CVD.

##### Preparation of CIF:FeNi, CIF:FeNiMo‐I, CIF:FeNiMo‐II, CIF:FeNiMo‐III, and CIF:NiMo

First, CIFs on nickel foam was cut (1 cm × 5 cm), and precursor solutions of 10 × 10^−3^ m FeCl_3_ and MoCl_5_ in pure ethanol were prepared. Then, two solutions were poured with different ratios in a conical tube (FeNi: FeCl_3_ 16 mL, FeNiMo‐I: FeCl_3_ 16 mL + MoCl_5_ 8 mL, FeNiMo‐II: FeCl_3_ 16 mL + MoCl_5_ 14 mL, FeNiMo‐III: FeCl_3_ 16 mL + MoCl_5_ 20 mL, and NiMo: MoCl_5_ 20 mL). The total volume of the solution was kept in 36 mL using pure ethanol. Subsequently, the cut CIFs on nickel foam was placed in the conical tube and was kept for 5 h at 60 °C. The as‐synthesized samples were washed with pure ethanol three times. Finally, all samples were dried overnight in vacuum oven at 60 °C.

##### Characterizations

Powder X‐ray diffraction patterns were obtained by a SmartLab X‐ray diffractometer (Rigaku, Japan) with Cu K*α* radiation at 1200 W (40 kV, 30 mA). X‐ray absorption spectroscopy including XANES and the EXAFS was conducted at a multipole‐wiggler 10C beamline and NEXAFS measurement were performed at the 4D beam line at the Pohang Accelerator Laboratory (PAL, Republic of Korea). The synchrotron radiations were monochromatized using a Si(111) double crystal monochromator. The incident beams were detuned with the proper rates for harmonic rejection. TEM and SEM images and video were obtained using a JEM‐ARM200F (JEOL, Japan) operated at 200 kV and JEM‐7600F (JEOL, Japan) operated at 15 kV, respectively. XPS spectra were collected by a Thermo VG Scientific K‐alpha (Thermo Scientific, USA) using Al K*α* radiation at 350 W (3 mA). Four‐point probe measurement was conducted by CMT‐SR2000 (Changmin Tech Co., Ltd).

##### Electrochemical Measurement

All electrochemical measurements were performed with a potentiostat (SP‐240, Biologic Science Instrument, USA) in 1 m KOH at room temperature. A three‐electrode system was used with the Hg/HgO (filled with 1 m KOH) and the Pt wire as reference and counter electrode, respectively. All samples were cut into a square shape (1 cm × 1 cm). The linear weep voltammetry (LSV) was carried out with scan rate of 1 mV s^–1^. All polarization curves were *iR* corrected at 95% *iR* compensation, where *R* is the series resistance measured by electrochemical impedance spectroscopy (EIS). The potentials were converted to the RHE potential using the Nernst equation (*E*_RHE_ = *E*_(Hg/HgO)_ + *E* ^0^_(Hg/HgO)_ + 0.0591*pH).

## Conflict of Interest

The authors declare no conflict of interest.

## Supporting information

Supporting Information

## Data Availability

Research data are not shared.
