# Supplementary material for: Metal‐Organic Fragments with Adhesive Excipient and Their Utilization to Stabilize Multimetallic Electrocatalysts for High Activity and Robust Durability in Oxygen Evolution Reaction
Source: Adv Sci (Weinh). 2021 Mar 24;8(11):2100044. doi: 10.1002/advs.202100044 (PMC8188218; doi:10.1002/advs.202100044)
Supplement: Supplementary file 1 — Supporting Information [file ADVS-8-2100044-s001.pdf]

WILEY-VCH

© 2021 Wiley-VCH GmbH

ADVANCED SCIENCE
Open Access

Supporting Information

for *Adv. Sci.*, DOI: 10.1002/advs.202100044

Metal-Organic Fragments with Adhesive Excipient and Their Utilization to Stabilize Multimetallic Electrocatalysts for High Activity and Robust Durability in Oxygen Evolution Reaction

Won Ho Choi, Keon-Han Kim, Heebin Lee, Jae Won Choi, Dong Gyu Park, Gi Hwan Kim, Kyung Min Choi, and Jeung Ku Kang**

Copyright © 2021 Wiley-VCH GmbH

Supporting Information

Metal-organic fragments with adhesive excipient and their utilization to stabilize multimetallic electrocatalysts for high activity and robust durability in oxygen evolution reaction

Won Ho Choi, Keon-Han Kim, Heebin Lee, Jae Won Choi, Dong Gyu Park, Gi Hwan Kim, Kyung Min Choi, and Jeung Ku Kang**

Dr. W. H. Choi, K. -H. Kim, H. Lee, Dr. J. W. Choi, D. G. Park, G. H. Kim, Prof. J. K. Kang
Department of Materials Science and Engineering
Korea Advanced Institute of Science and Technology (KAIST)
291 Daehak-ro, Yuseong-gu, Daejeon 34141, Republic of Korea
*Email: jeungku@kaist.ac.kr

Prof. K. M. Choi
Department of Chemical and Biological Engineering
Sookmyung Women's University
Cheongpa-ro 47-gil 100, Yongsan-gu, Seoul 04310, Republic of Korea
*Email: kmchoi@sookmyung.ac.kr

Section S1. *Supplementary figures*

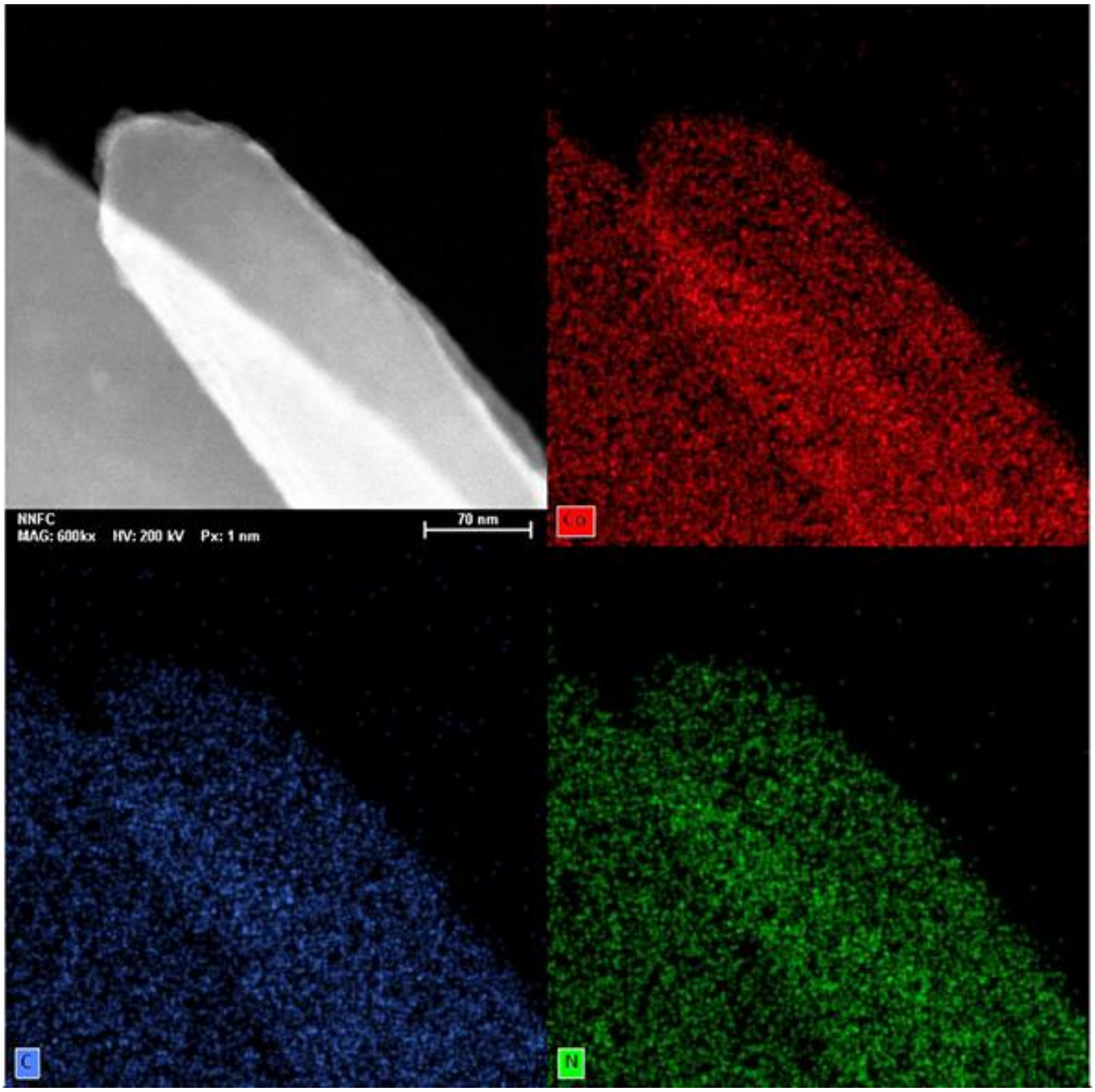

Figure S1. HADDF-STEM and EDS images of ZIF-L. The red, blue, and green represent cobalt, carbon, and nitrogen, respectively.

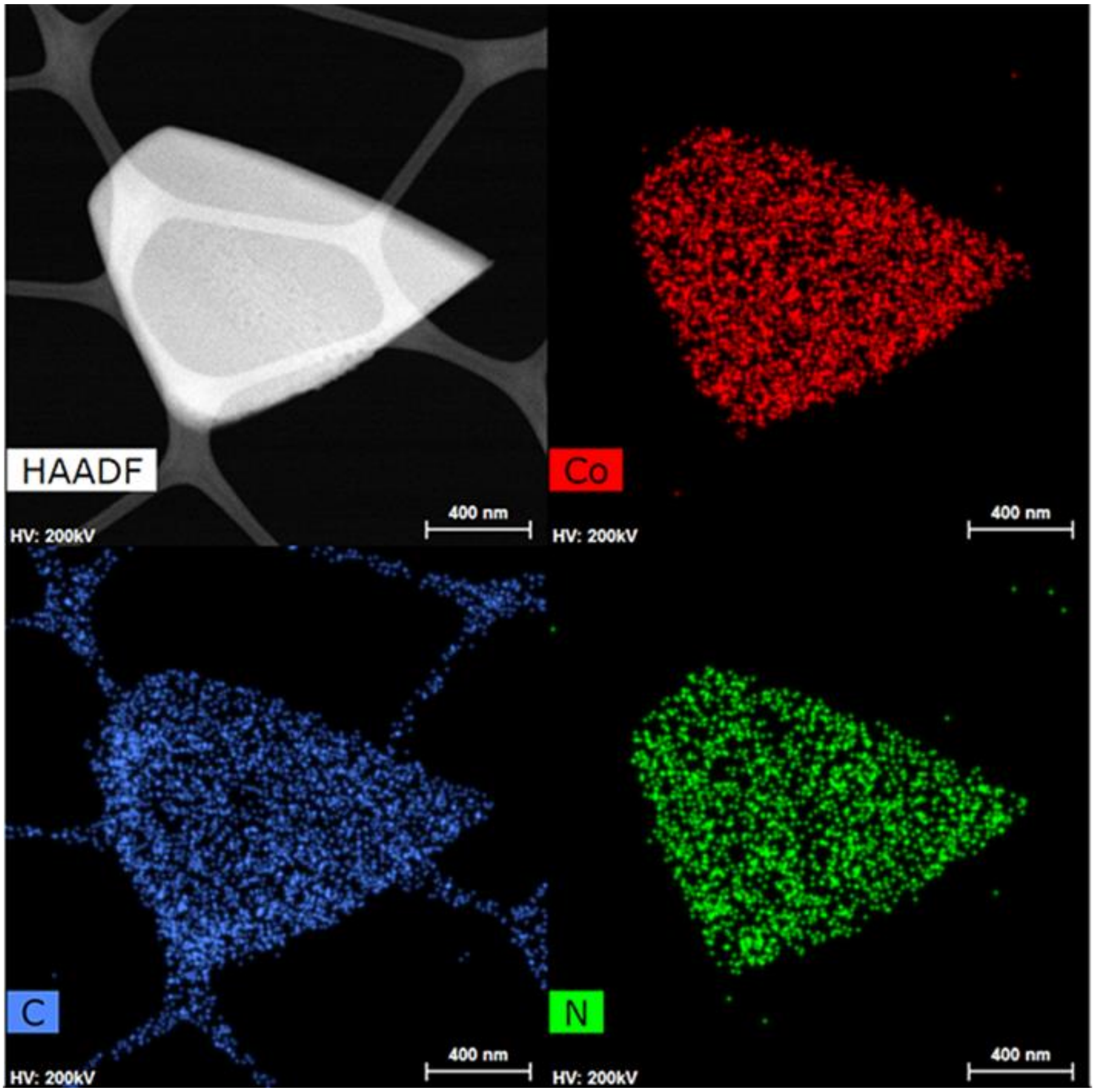

Figure S2. HADDF-STEM and EDS images of CIF. The red, blue, and green represent cobalt, carbon, and nitrogen, respectively.

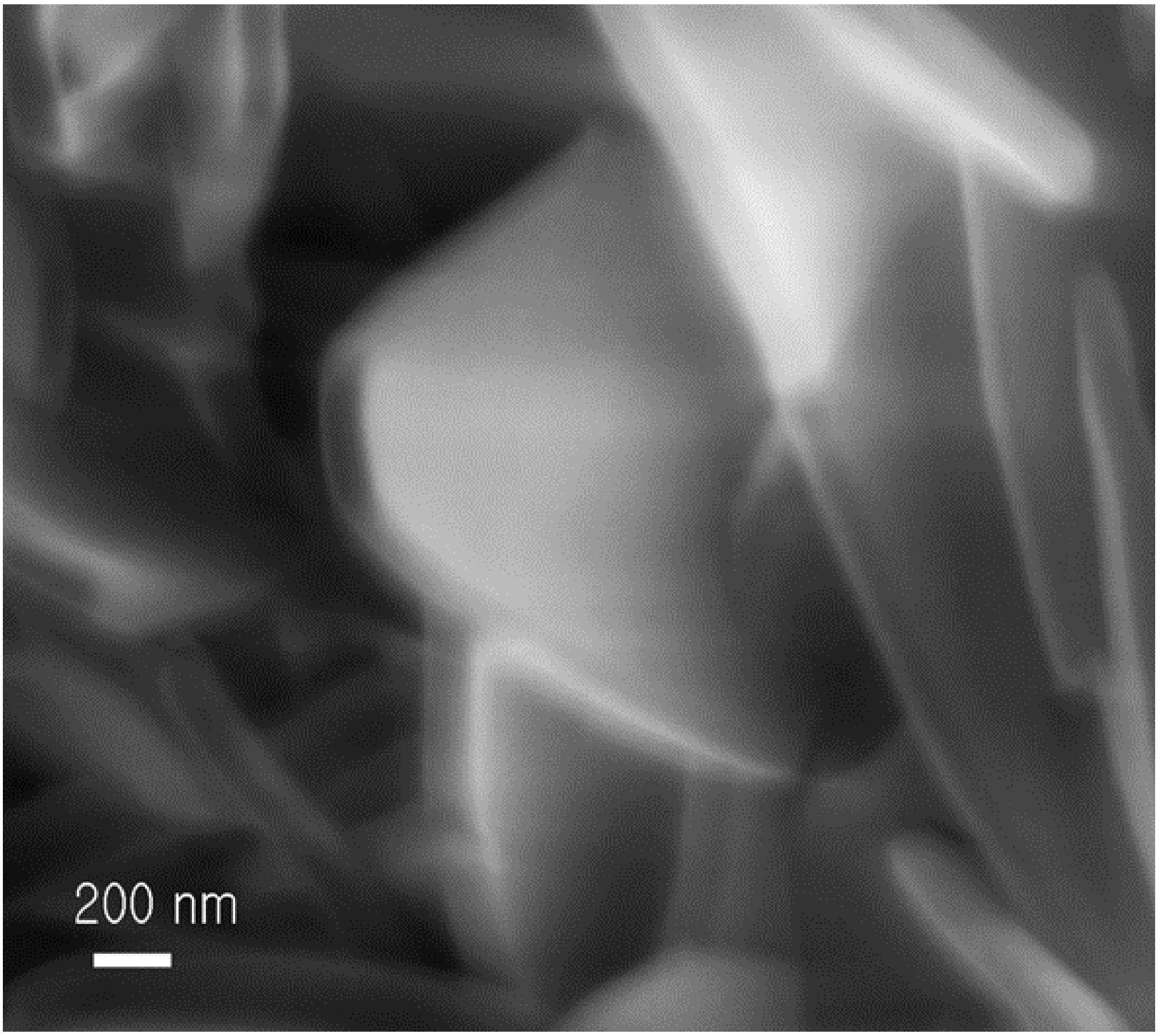

Figure S3. SEM image of Co-based ZIF-L after H_2 plasma treatment.

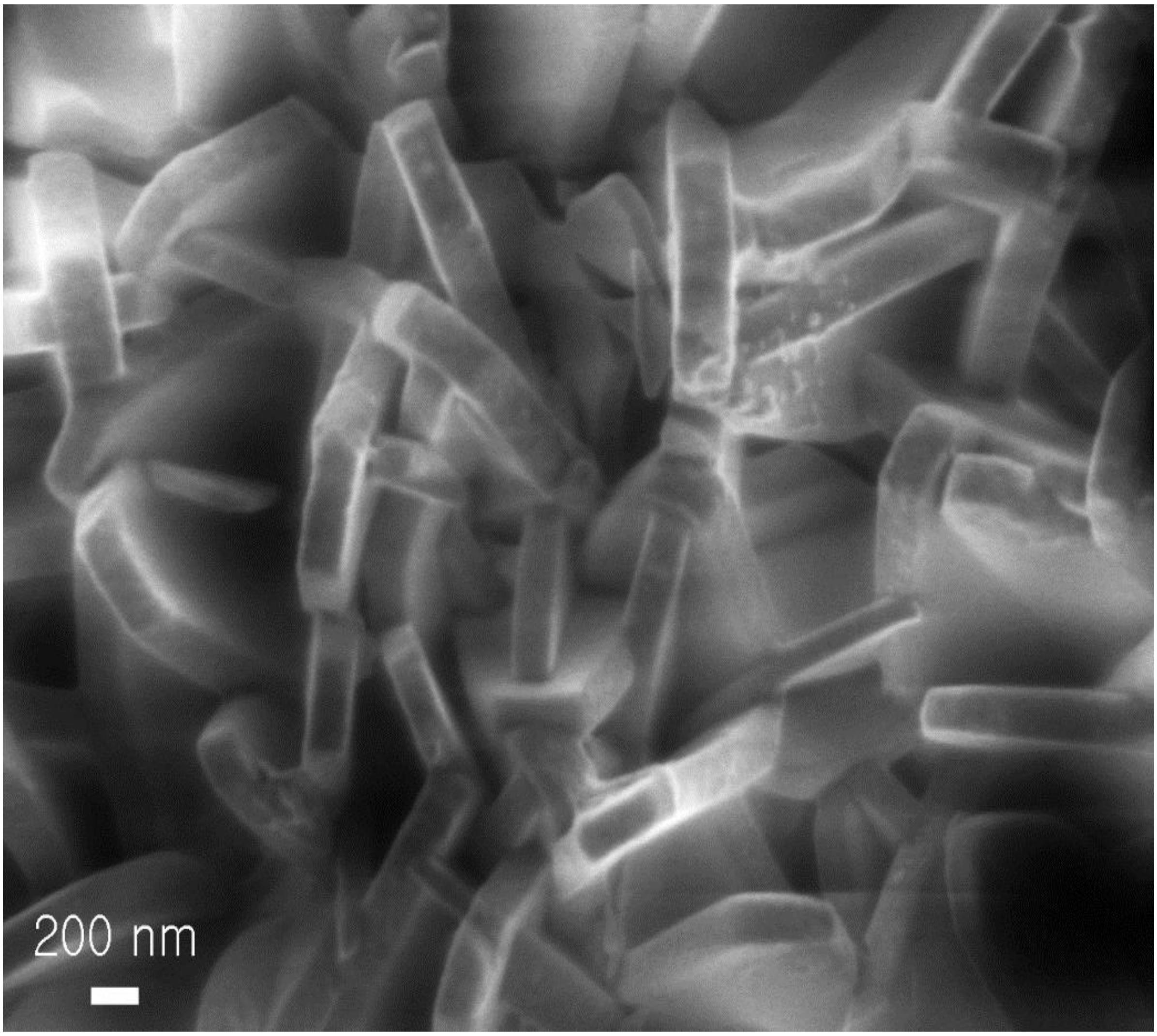

Figure S4. SEM image of Co-based ZIF-L after H_2 plasma treatment.

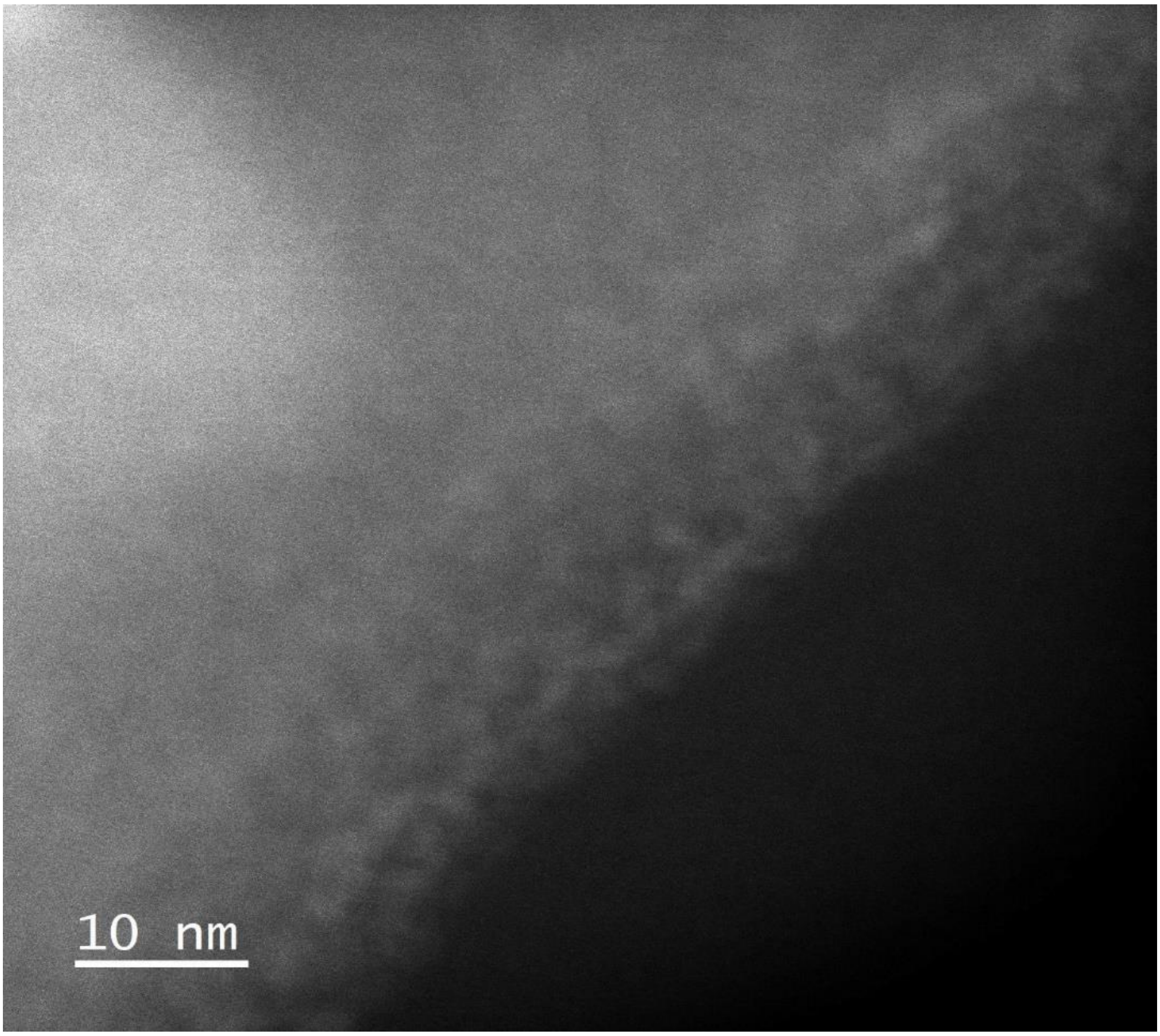

Figure S5. HR-TEM image of Co-based ZIF-L after H_2 plasma treatment.

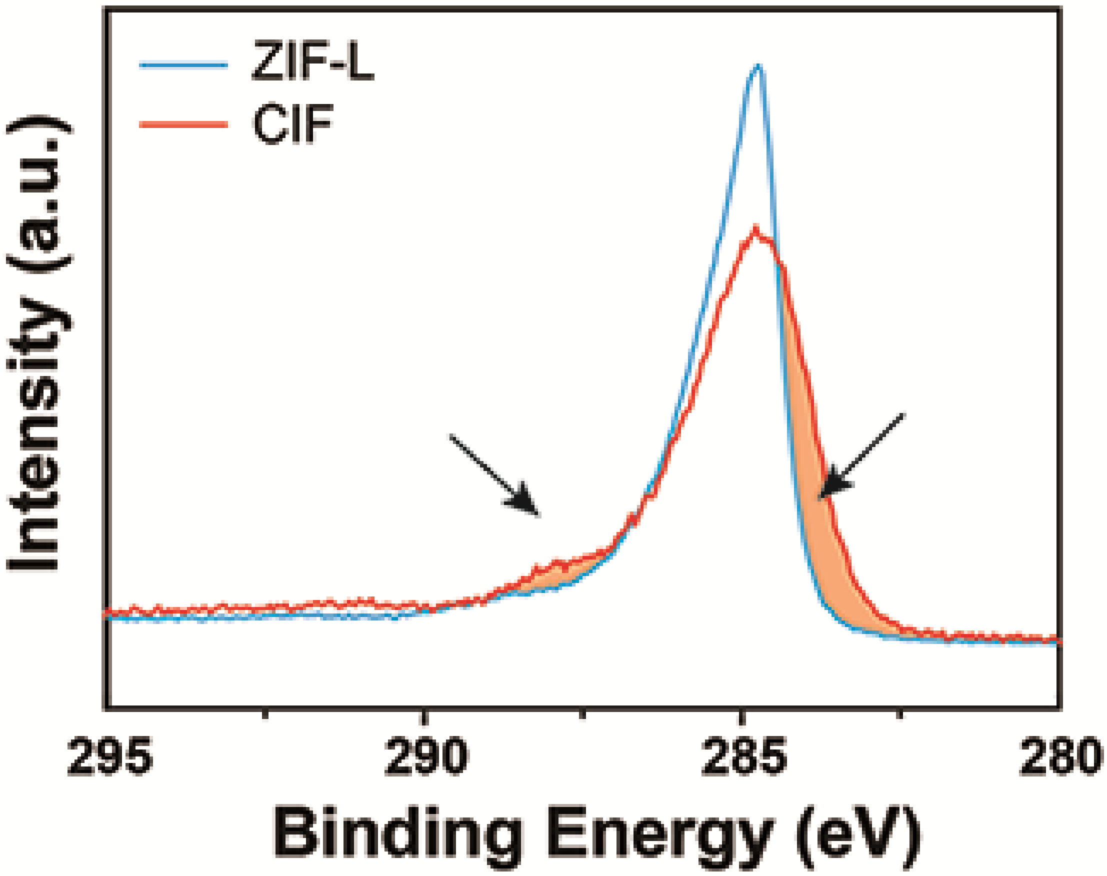

Figure S6. C 1s XPS spectra of ZIF-L and CIF.

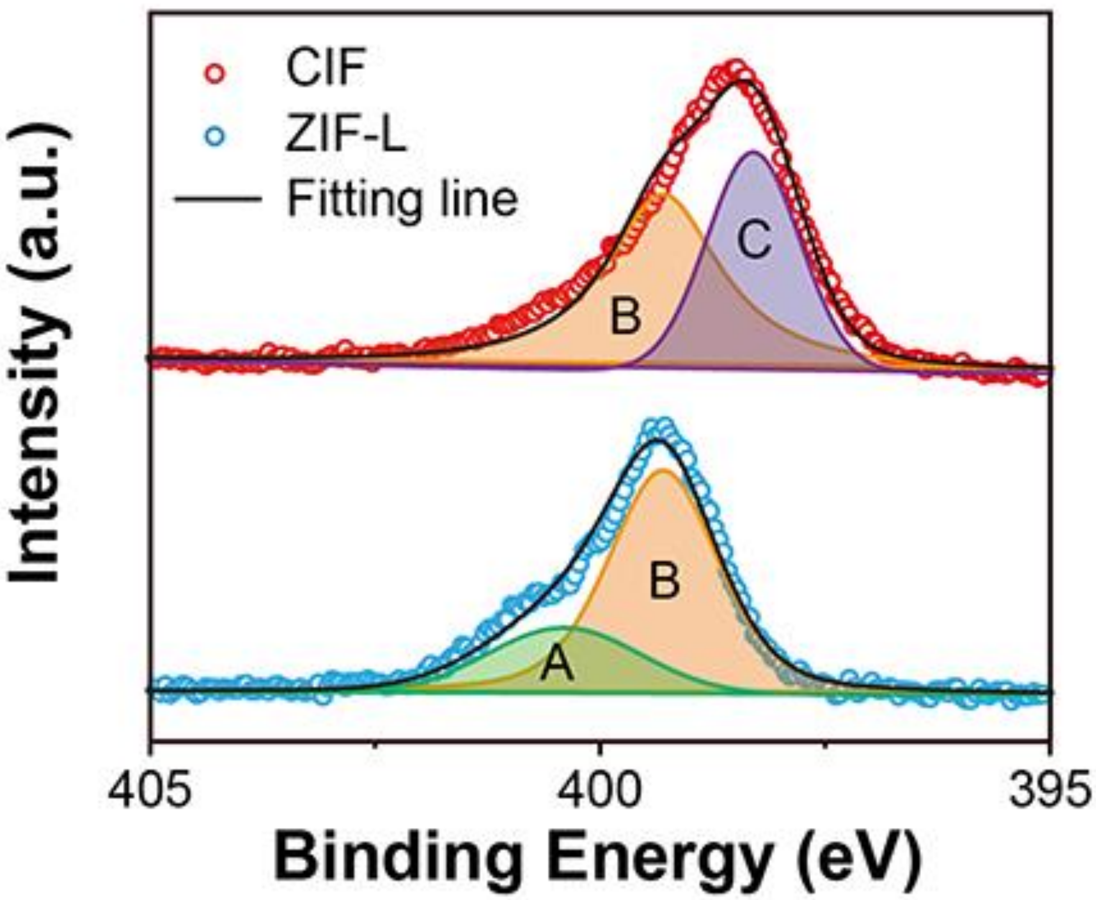

Figure S7. N 1s XPS spectra of ZIF-L and CIF. (A peak position: 400.4 eV, B peak position: 399.3 eV, and C peak position: 398.3 eV)

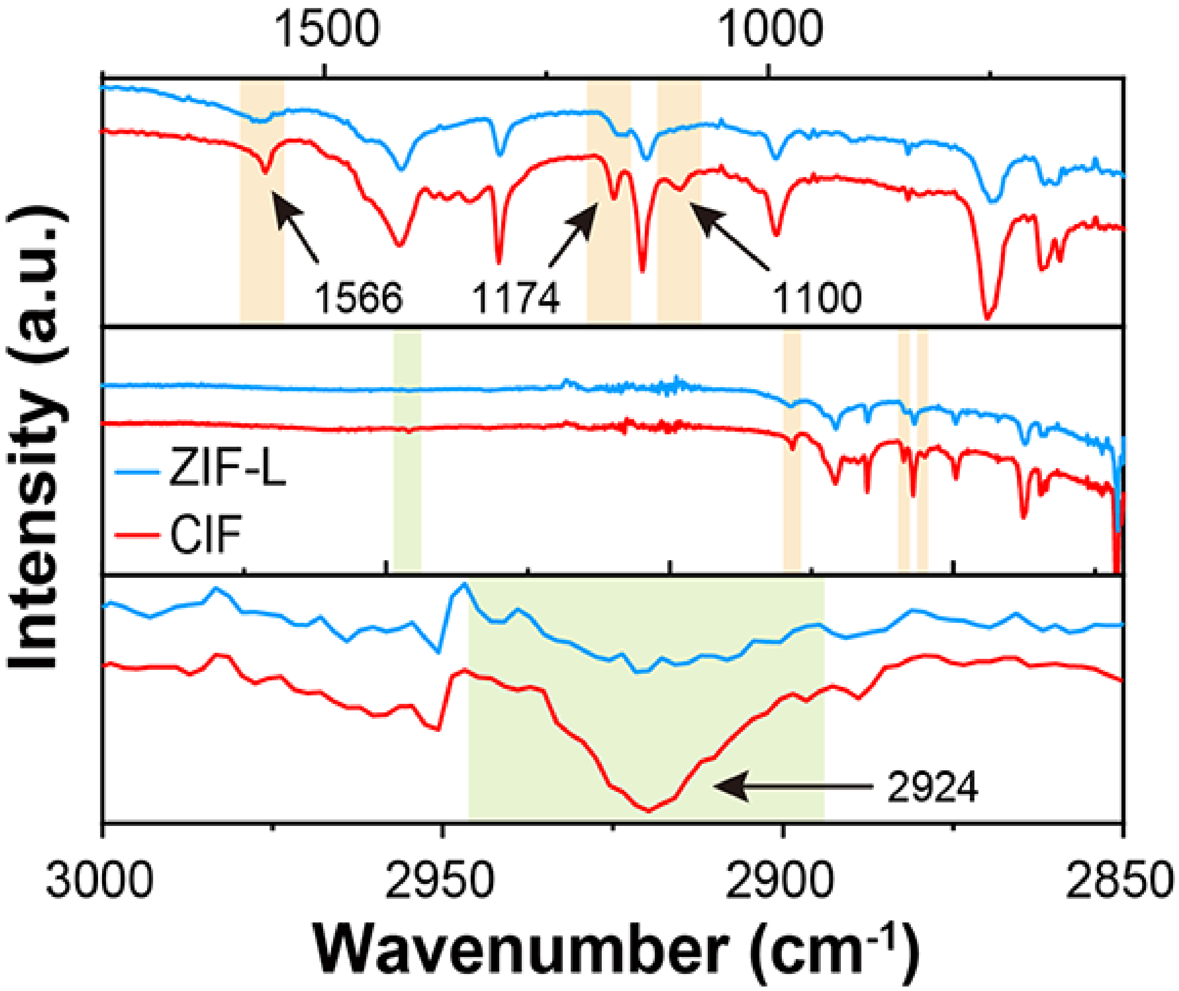

Figure S8. IR spectra of ZIF-L and CIF, The spectra in top and botoom are enlarged spectra of entire wavenumber(400~4000 cm^{-1}) of spectra in the middle.

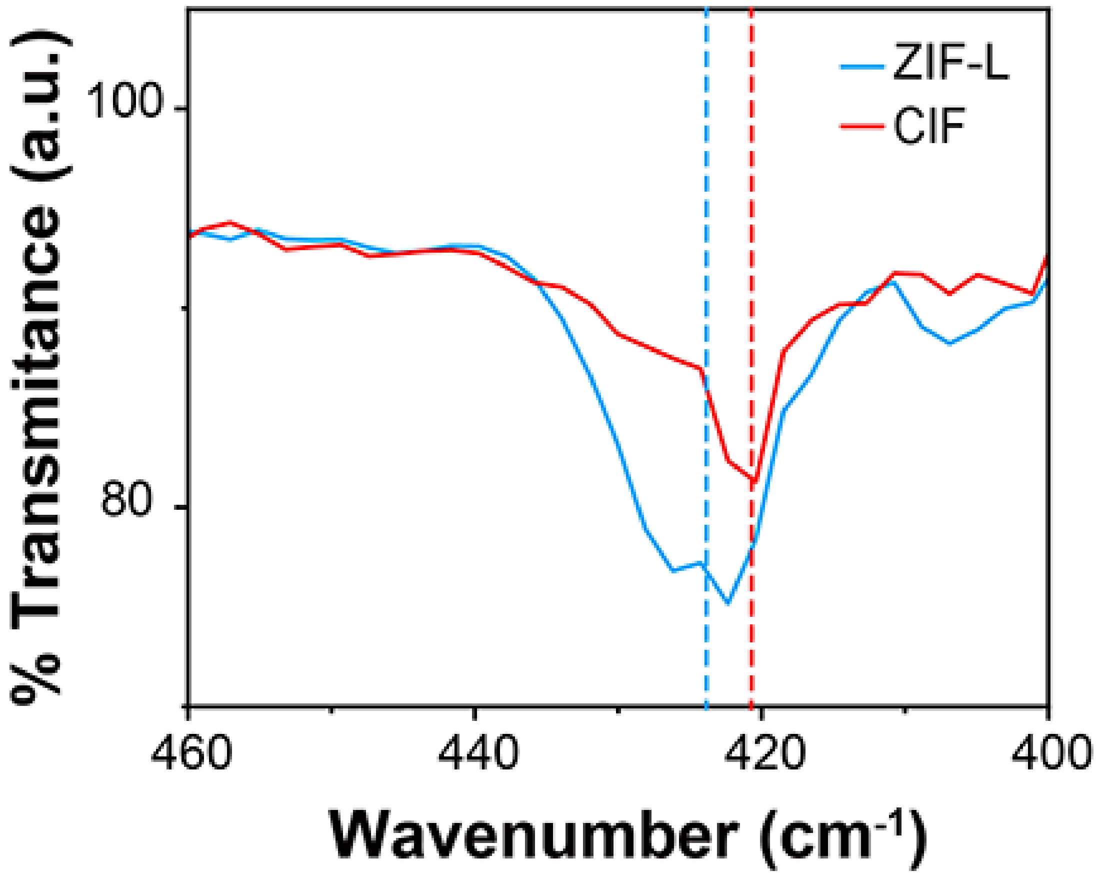

Figure S9. IR spectra corresponding to the fingerprint area.

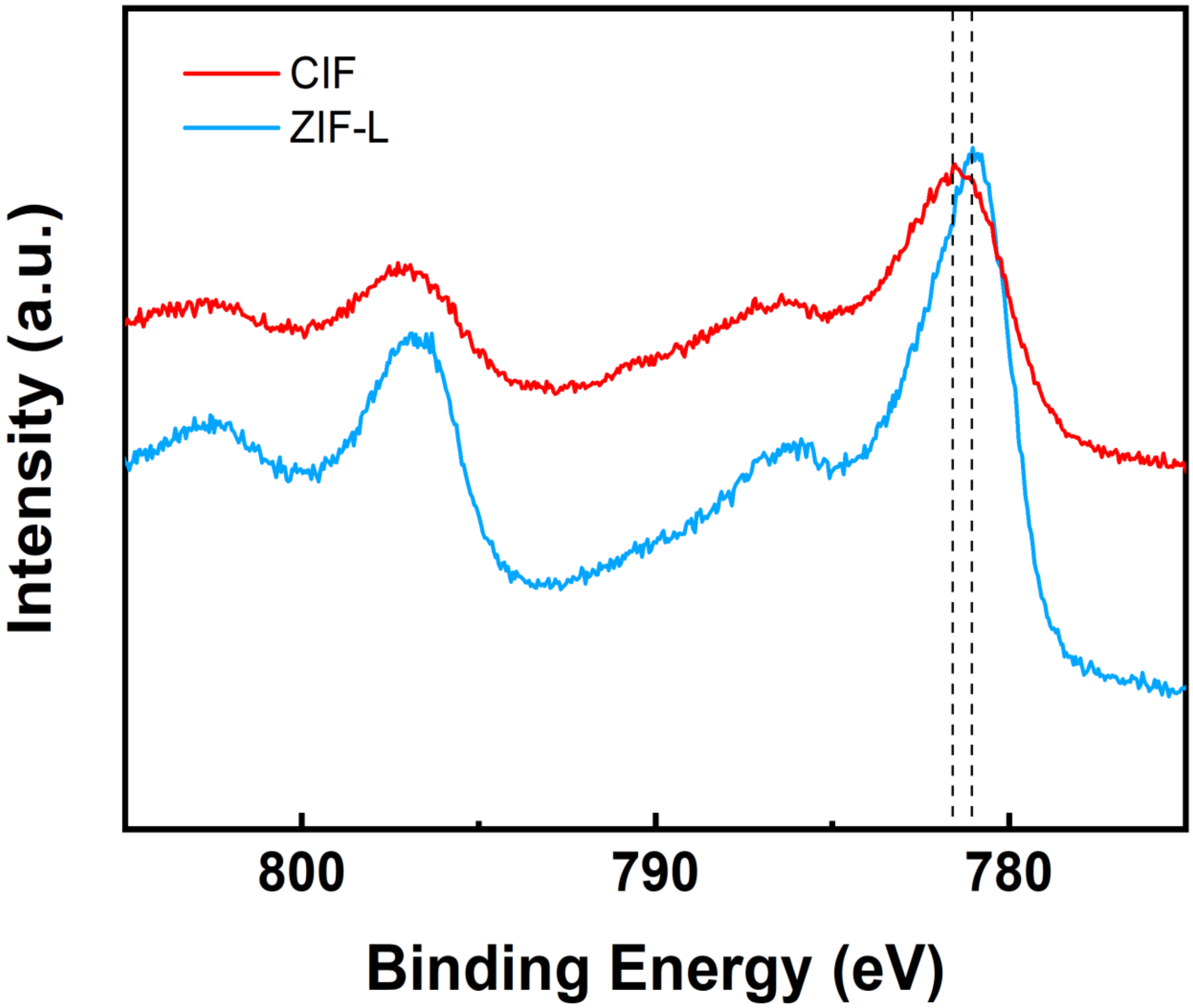

Figure S10. Co 2p XPS spectra of ZIF-L and CIF.

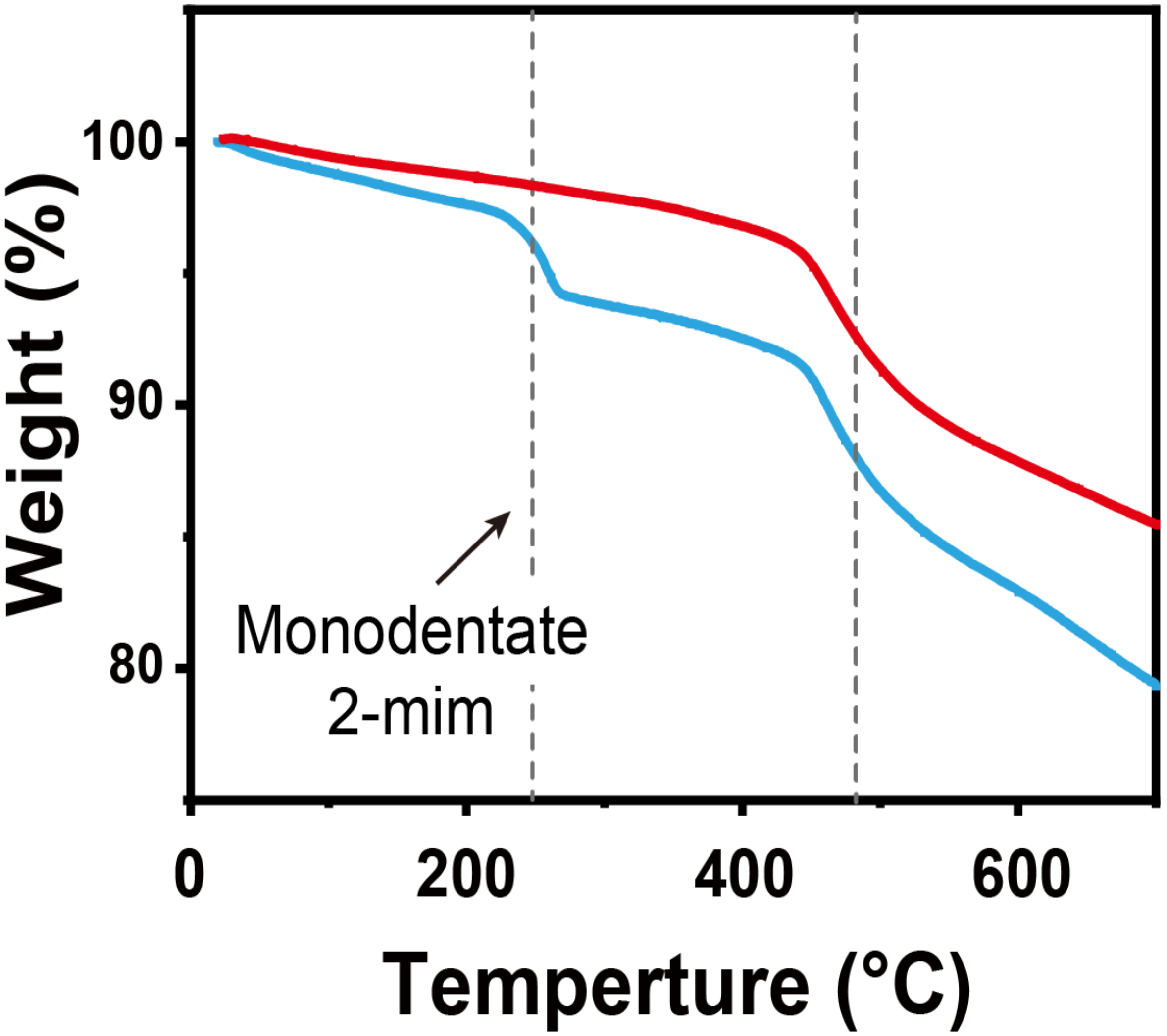

Figure S11. TGA curves at nitrogen atmosphere.

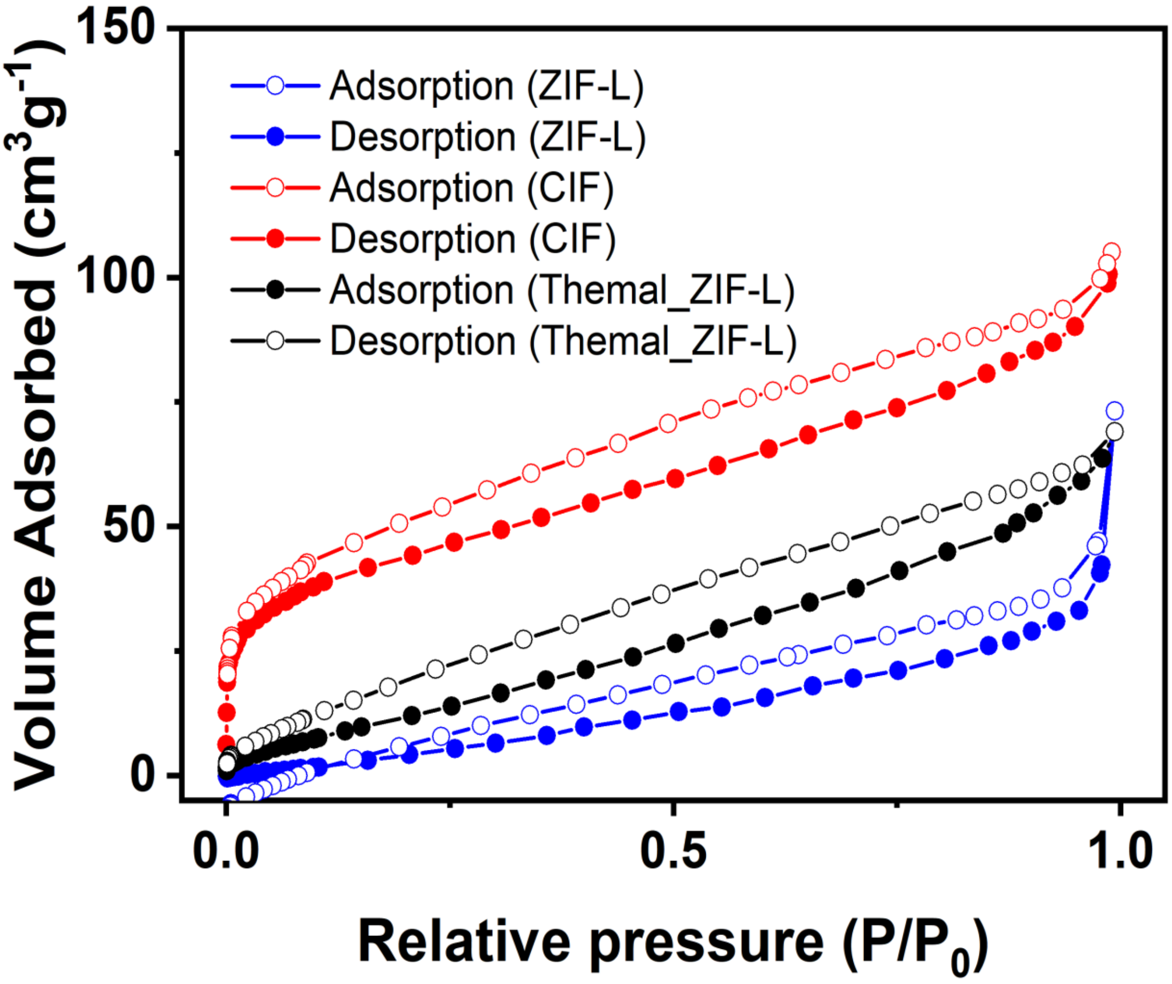

Figure S12. BET N_2 adsorption isotherms of ZIF-L, CIF, and ZIF-L (thermal treatment).

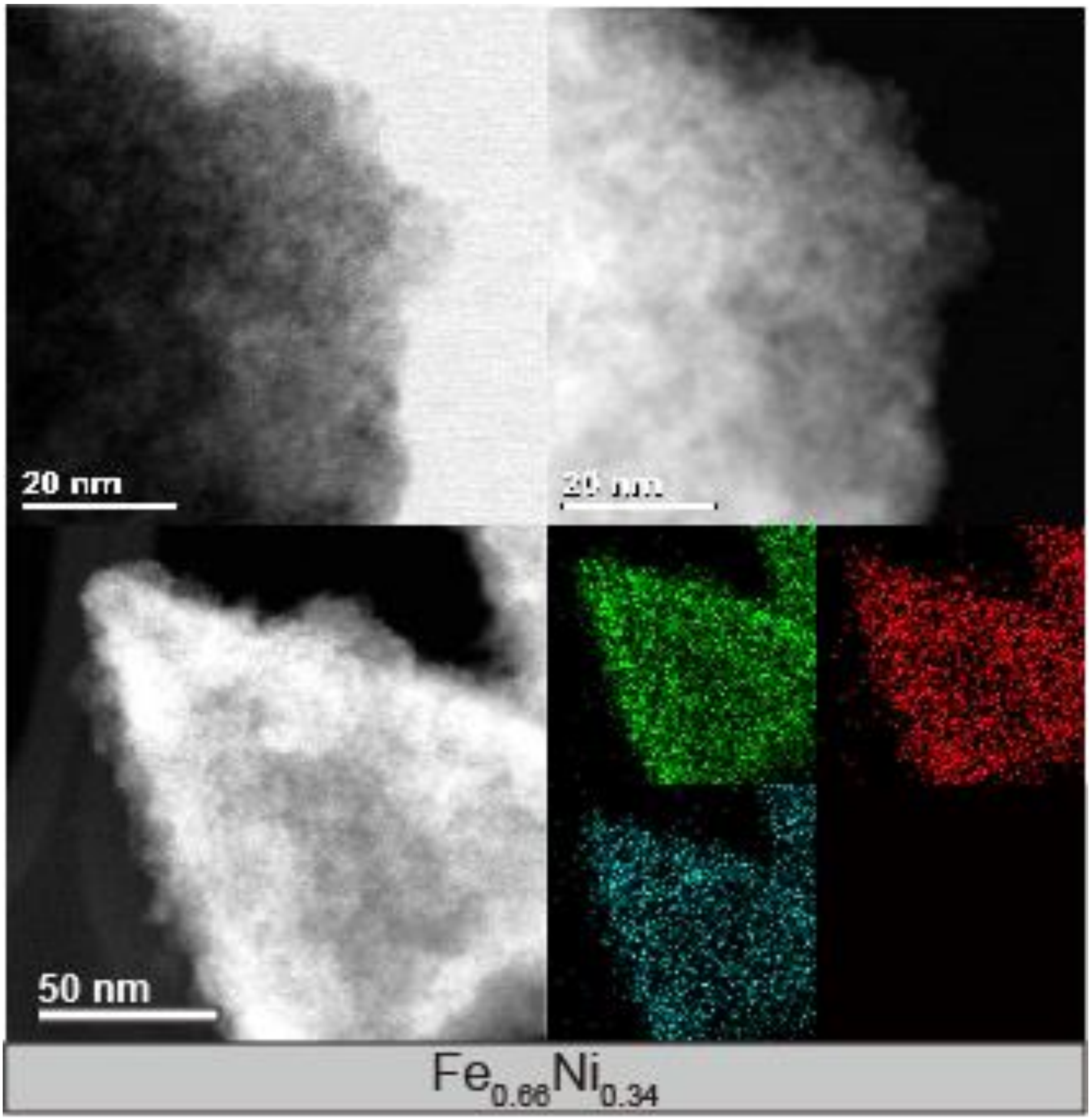

Figure S13. TEM, HADDF-STEM, and EDS images of CIF:FeNi. The green, red, and blue represent iron, cobalt, and nickel, respectively.

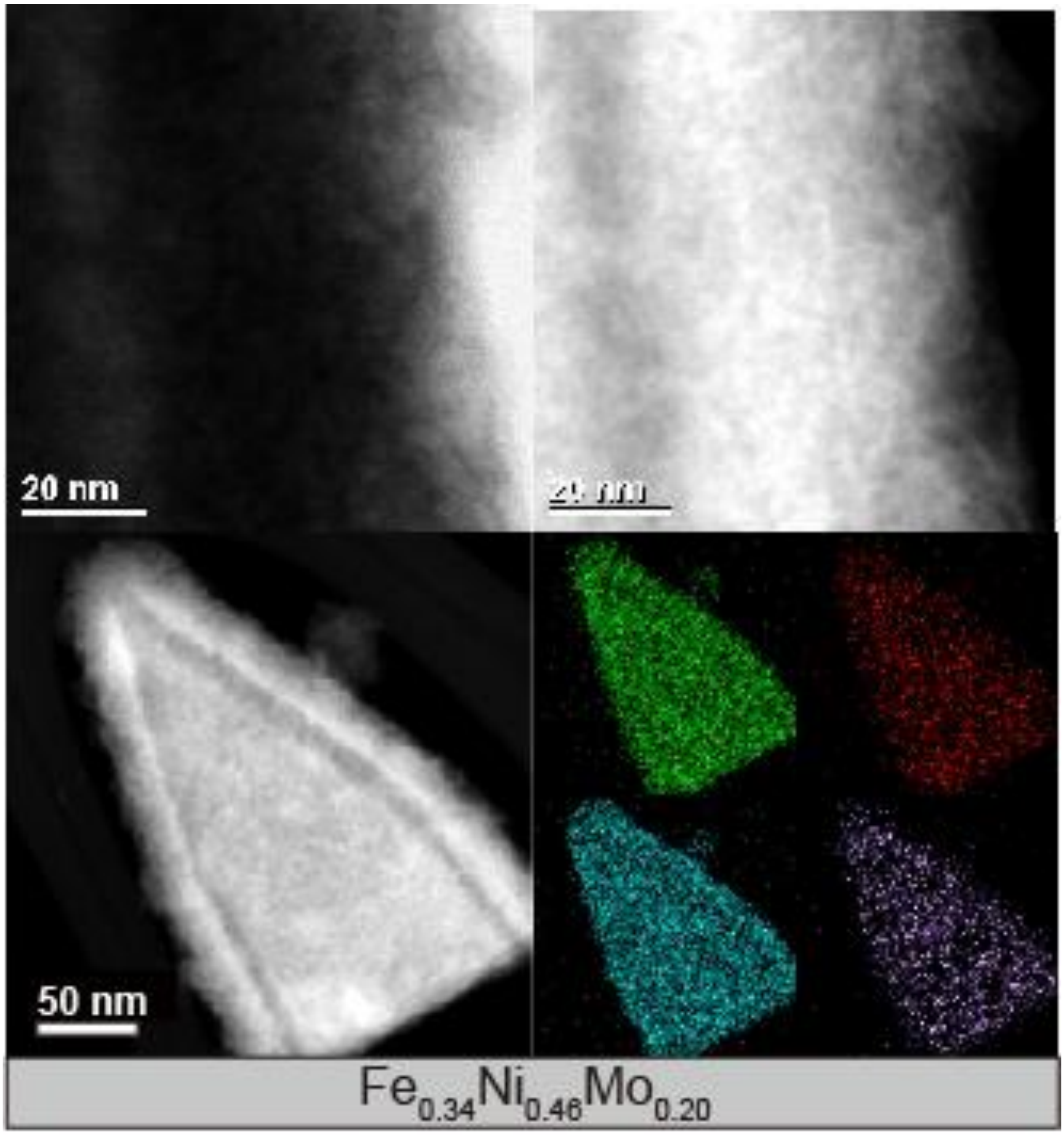

Figure S14. TEM, HADDF-STEM, and EDS images of CIF:FeNiMo-I. The green, red, blue, and purple represent iron, cobalt, nickel, and molybdenum, respectively.

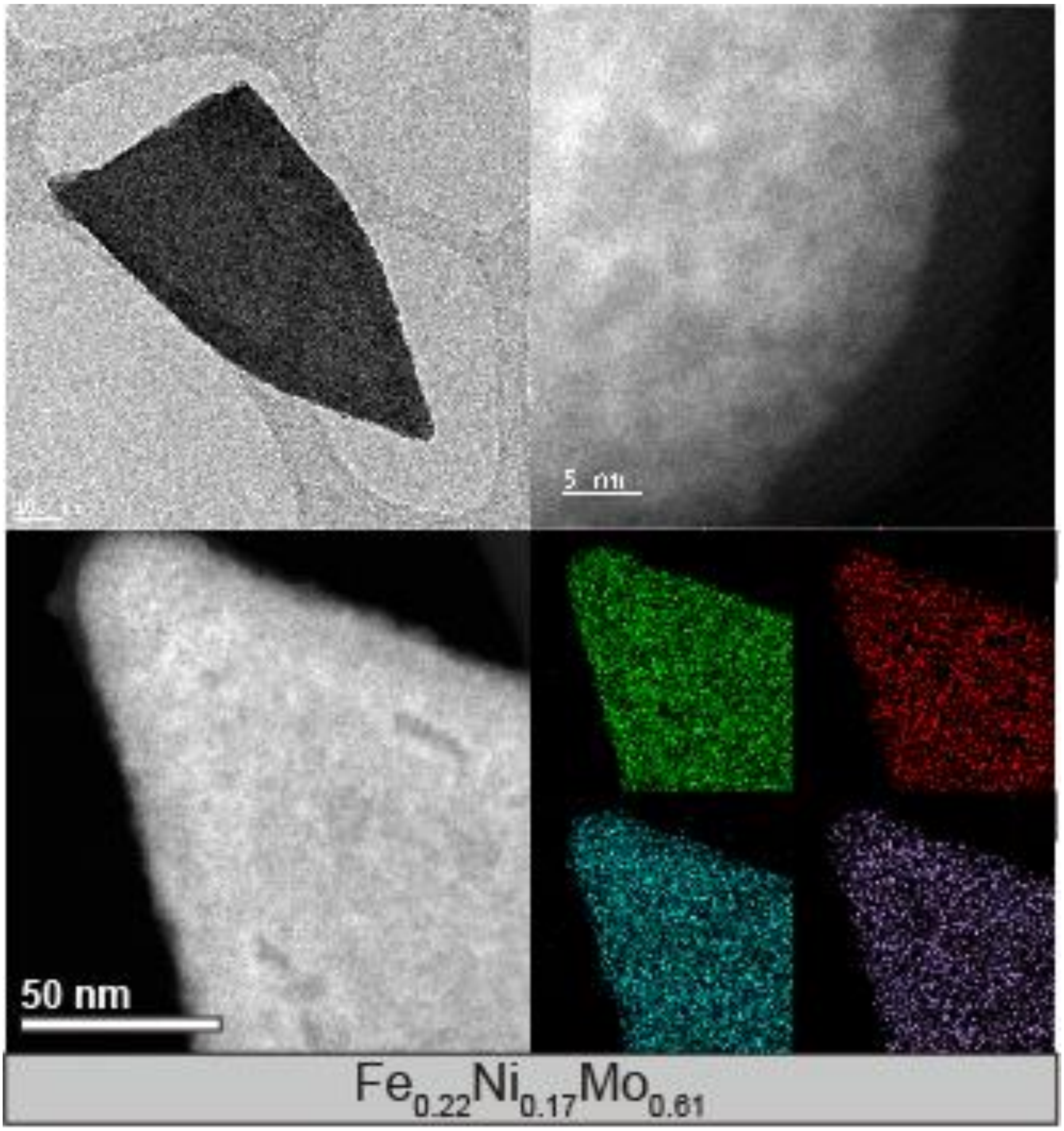

Figure S15. TEM, HADDF-STEM, and EDS images of CIF:FeNiMo-II. The green, red, blue, and purple represent iron, cobalt, nickel, and molybdenum, respectively.

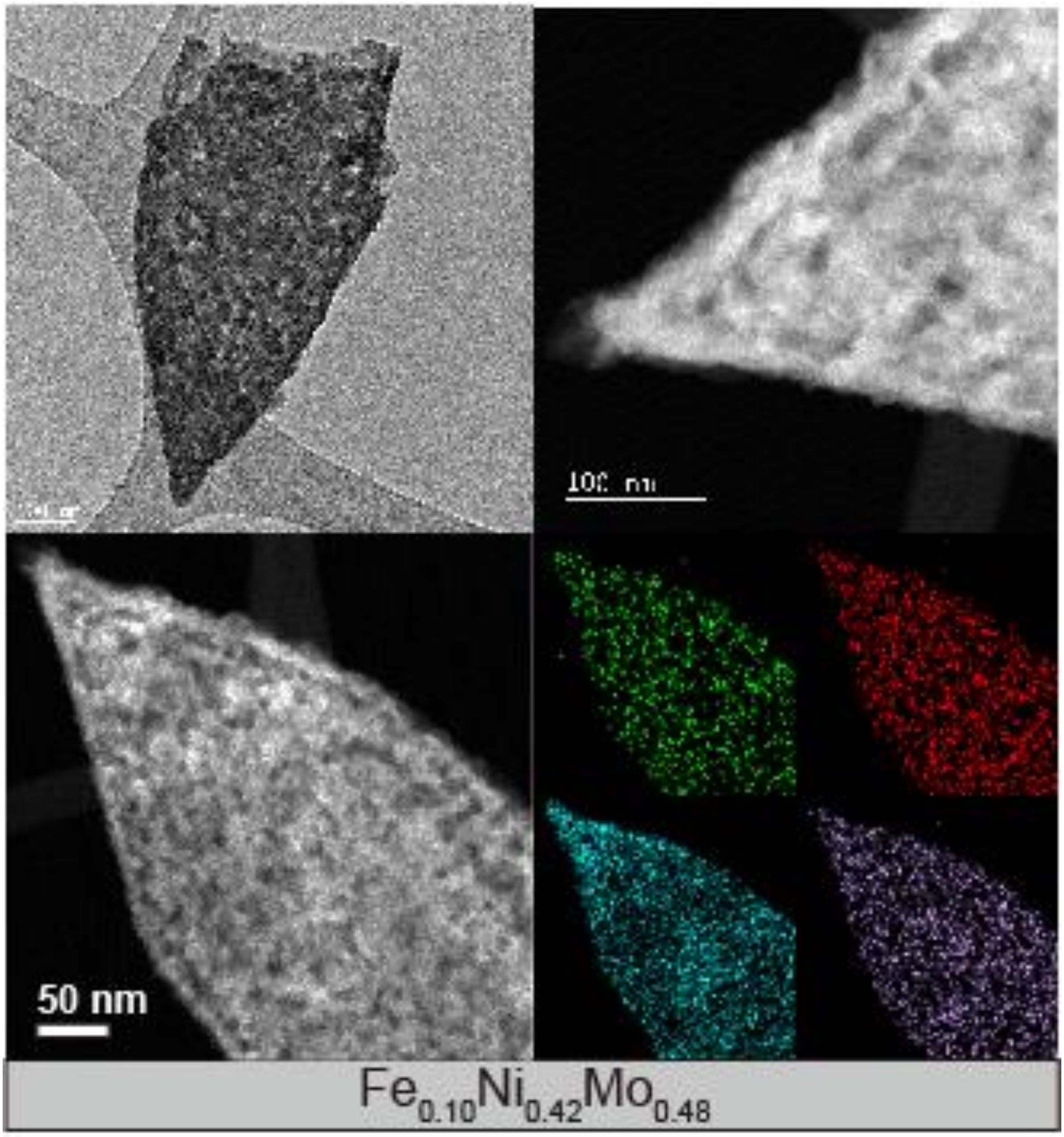

Figure S16. TEM, HADDF-STEM, and EDS images of CIF:FeNiMo-III. The green, red, blue, and purple represent iron, cobalt, nickel, and molybdenum, respectively.

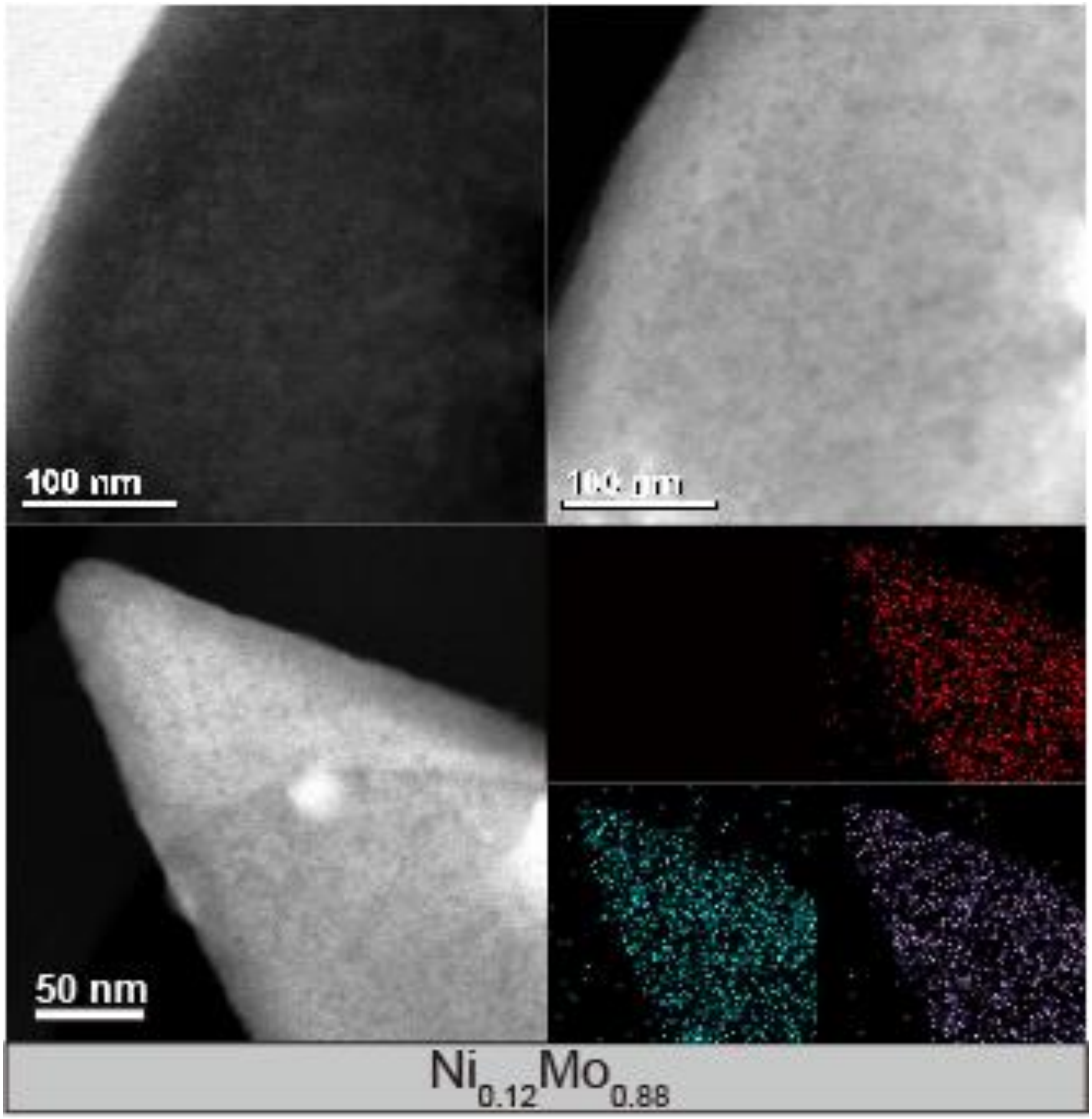

Figure S17. TEM, HADDF-STEM, and EDS images of CIF:NiMo. The red, blue, and purple represent cobalt, nickel, and molybdenum, respectively.

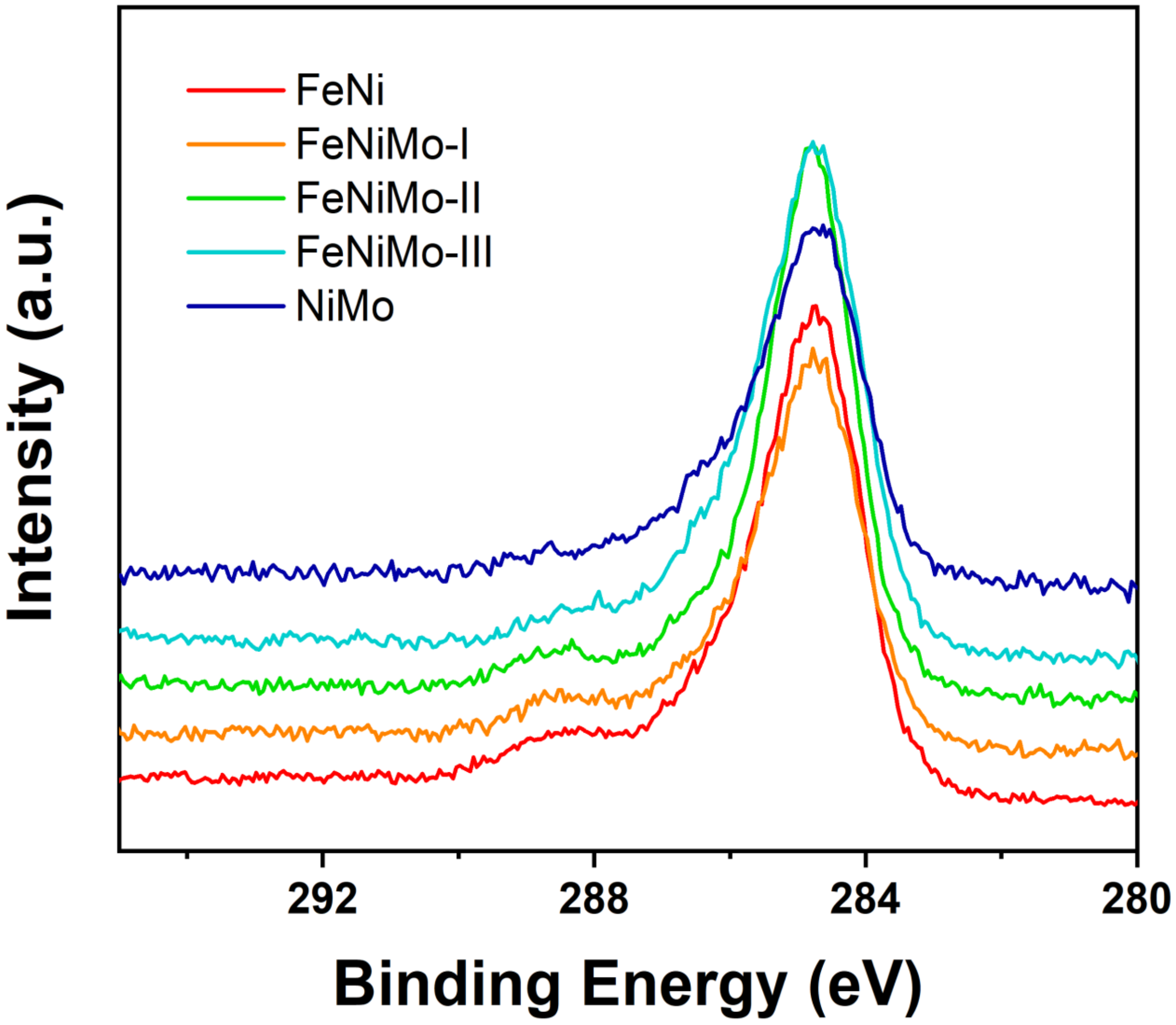

Figure S18. C 1s XPS spectra of CIF:FeNi, FeNiMo-I, FeNiMo-II, FeNiMo-III, and NiMo.

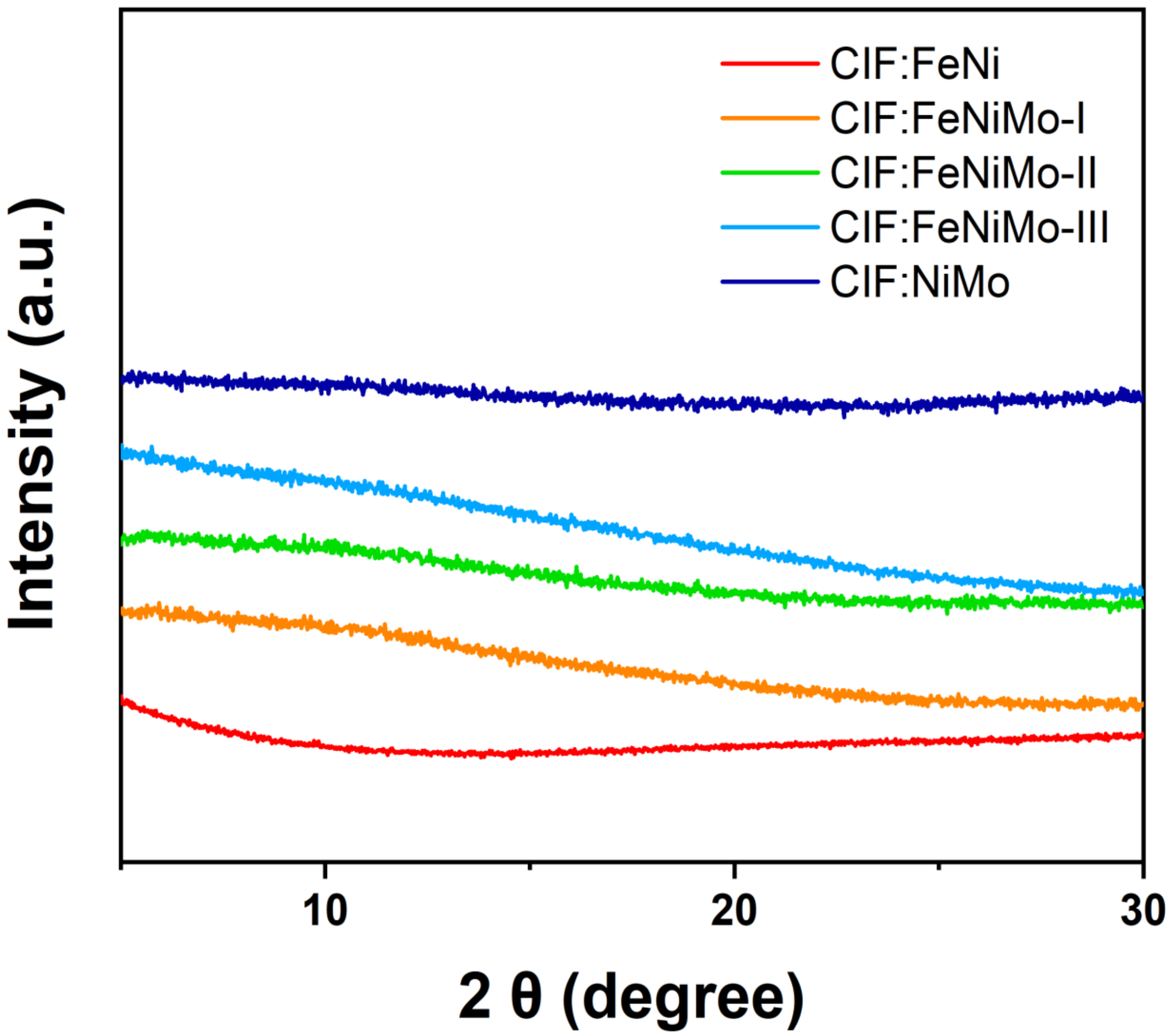

Figure S19. XRD patterns of CIF:FeNi, FeNiMo-I, FeNiMo-II, FeNiMo-III, and NiMo.

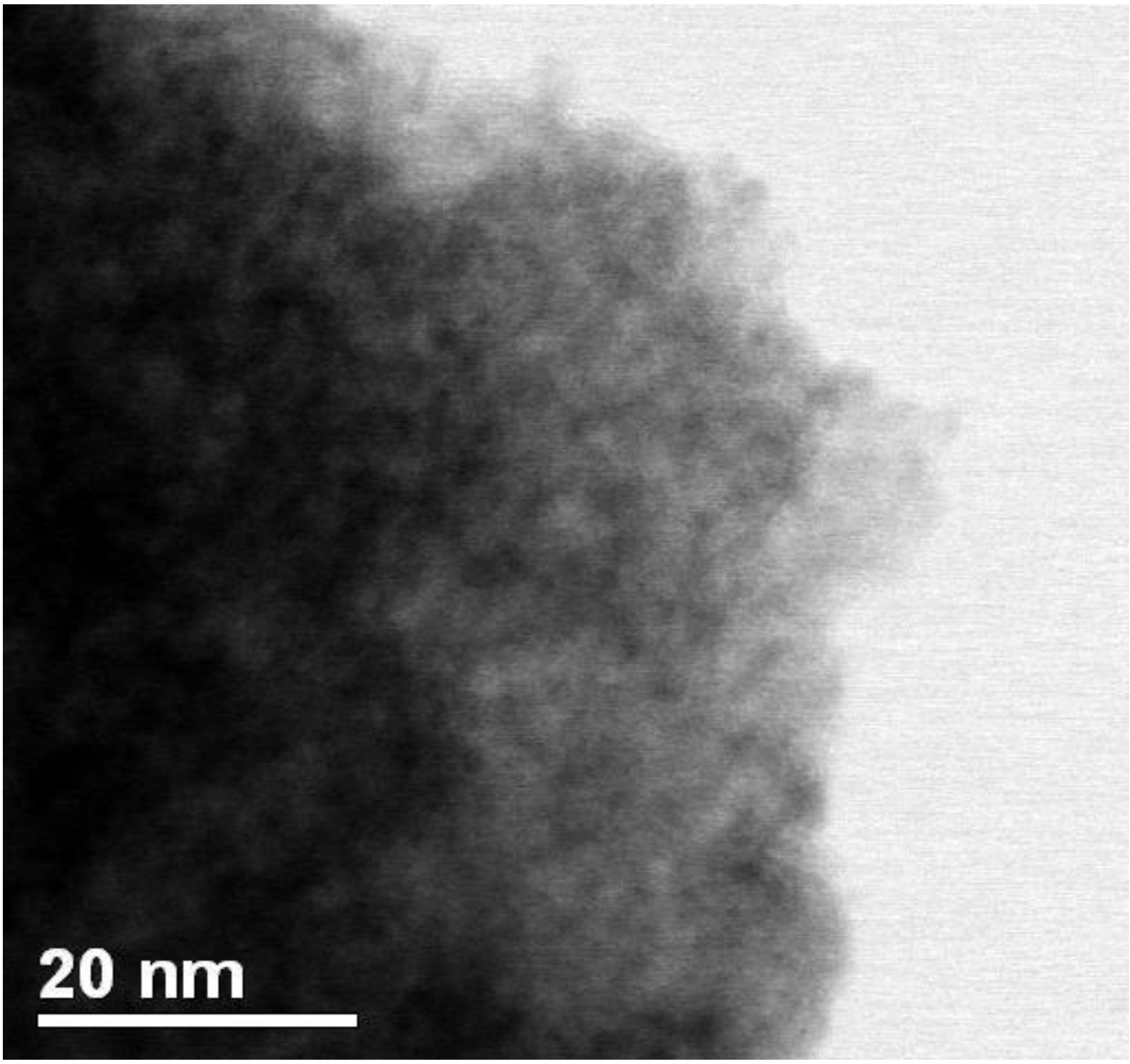

Figure S20. HR-TEM image of CIF:FeNi.

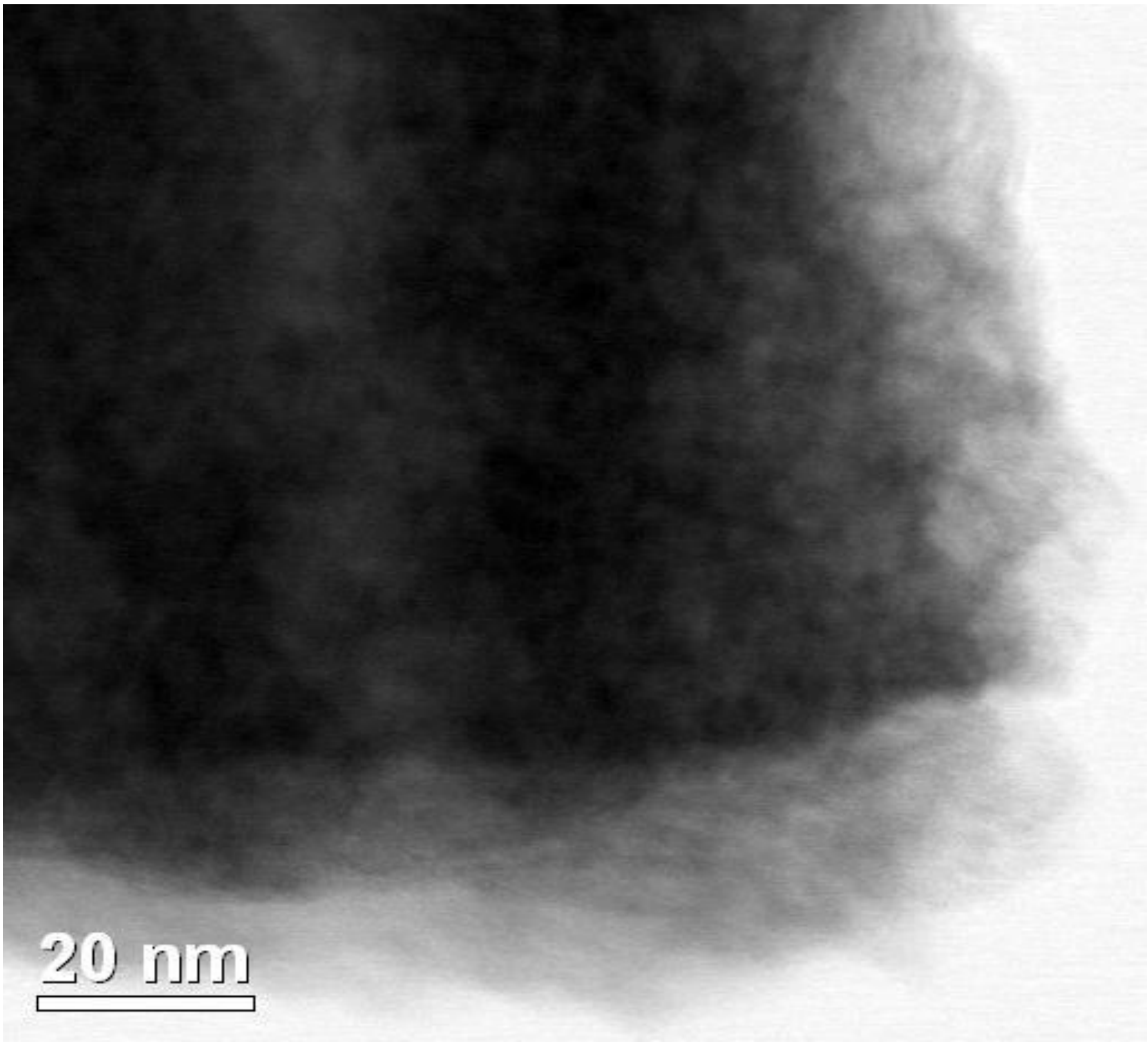

Figure S21. HR-TEM image of CIF:FeNiMo-I.

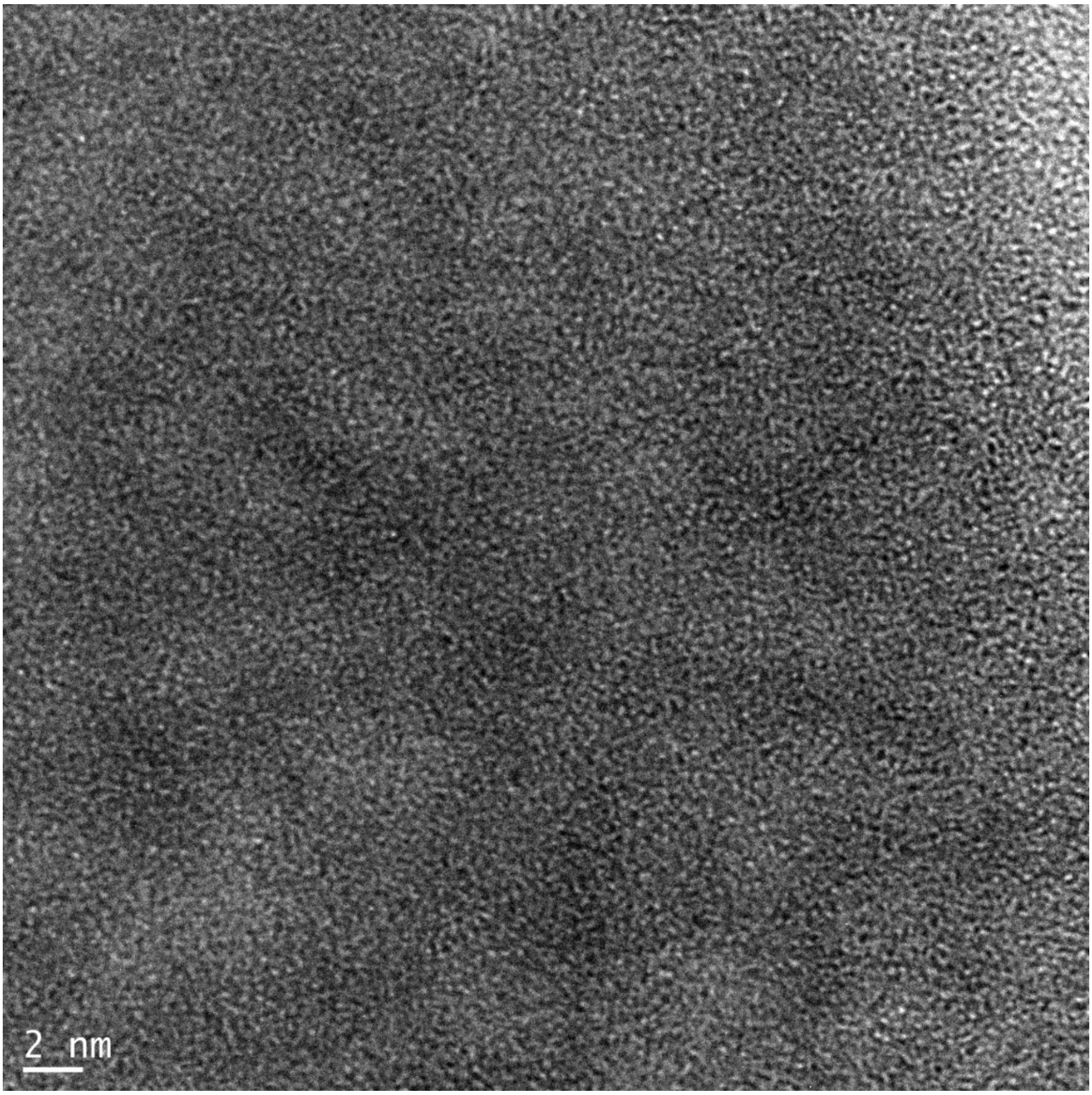

Figure S22. HR-TEM image of CIF:FeNiMo-II.

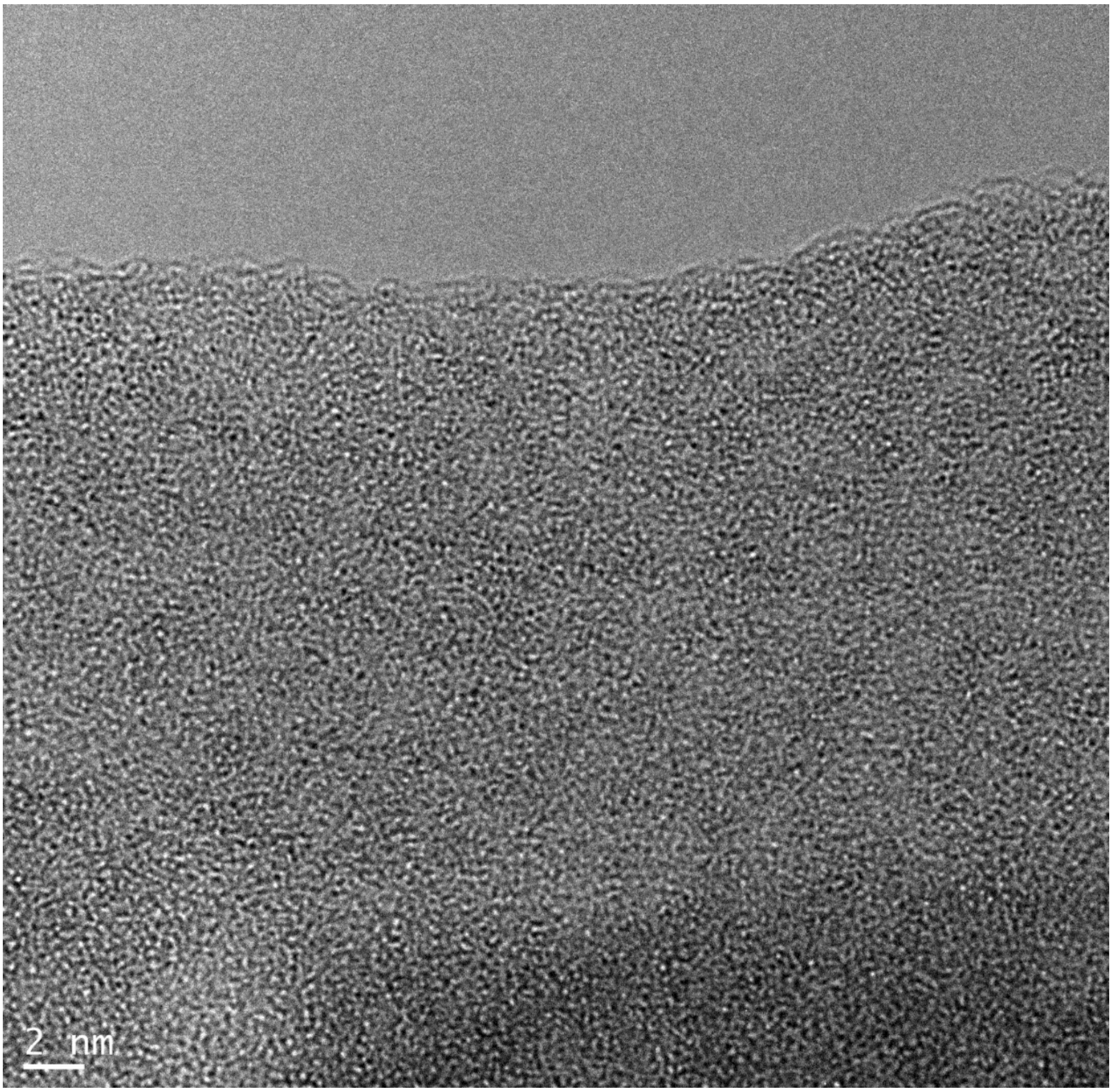

Figure S23. HR-TEM image of CIF:FeNiMo-III.

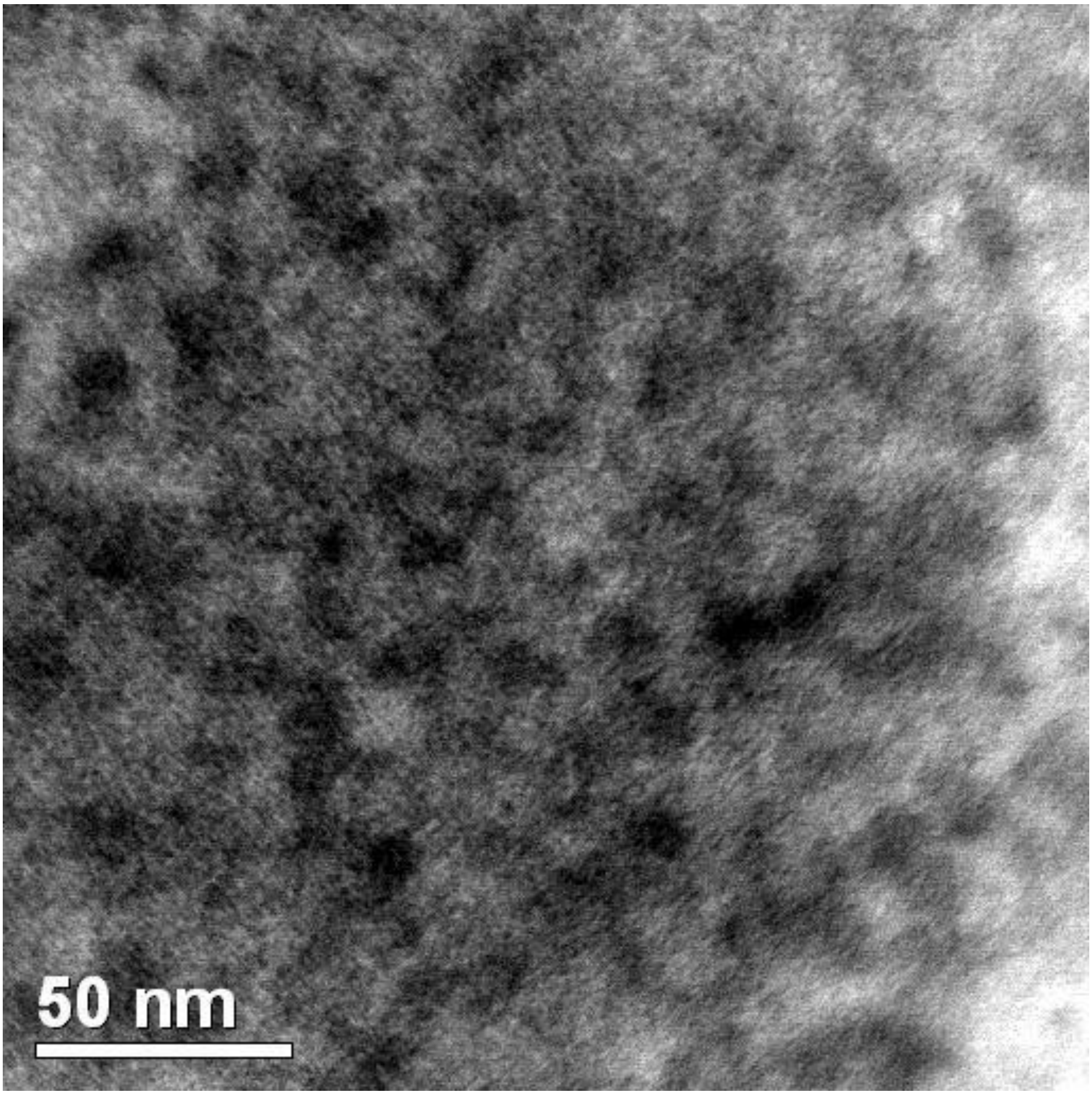

Figure S24. HR-TEM image of CIF:NiMo.

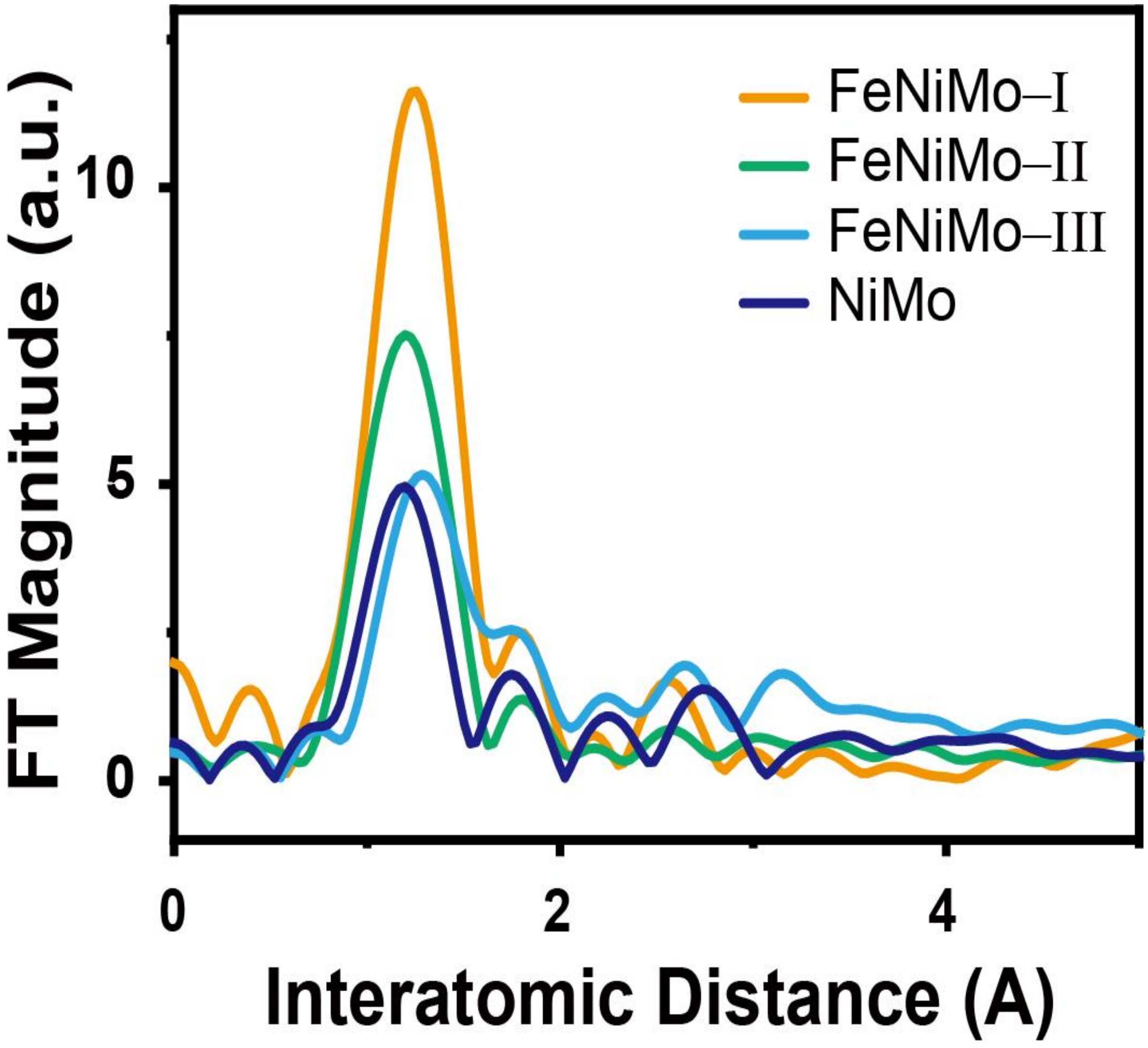

Figure S25. Radial distribution function obtained by the Fourier transformation of k3-weighted Mo EXAFS spectra of CIF:FeNi, FeNiMo-I, FeNiMo-II, FeNiMo-III, and NiMo.

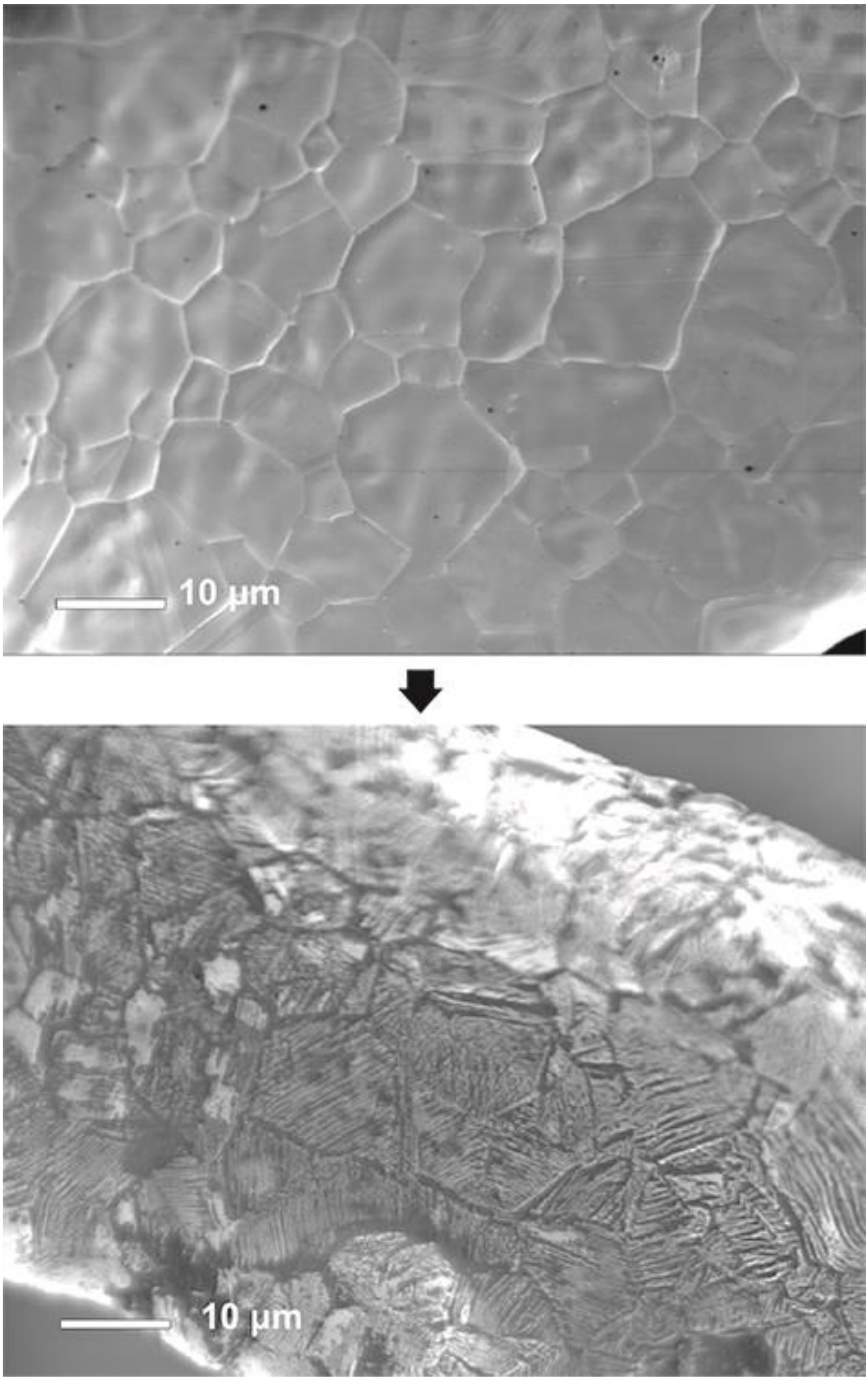

Figure S26. SEM image of NF (top) and NF:FeNiMo without CIF (bottom).

Section S2. *Supplementary tables*

Sample	Specific Surface Area (m^2 g^{-1})	Micropore Characteristics	
		Surface Area (m^2 g^{-1})	Pore Volume (cc/g)
ZIF-L	63.2	0.024	0.00
CIF	159.5	56.5	0.017

Table S1. Specific urface areas of ZIF-L and CIF along with their micropore characterisitics calculated using the BET isotherms and *t*-plot method.

Catalyst material	Precursor	Overpotential (mV at 10 mA cm^{-2})	Tafel slope (mV dec^{-1})	Durability (at least)	Ref.
FeNi	Prussian blue analogues	261	40	120 hours at 10 mA cm^{-2}	[44]
Ni_2P/CoN	ZIF-8/ZIF-67	270	65	2000 cycles at 1.55 V	[45]
FeNiP	PCN-600-Ni	240	63	3000 cycles at 1.47 V	[46]
$(Ni_{0.62}Fe_{0.38})_2P$	Prussian blue analogues	290	44	10 h at 1.53 V	[47]
$Cu_{0.3}Co_{2.7}P$	Cu doped ZIF-67	190	44	1000 cycles at 1.68 V	[48]
$FeNi_{2.4}Co_{0.4}$	MIL-53	219	53.5	1000 cycles at 1.53 V	[49]
FeNi	MIL-53	227	38.9	10 h at 10 mA cm^{-2}	[50]
CoNiP	ZIF-67	209	52	3000 cycles at 50 mV s^{-1}	[51]
FeNiMo	ZIF-L	203	34	45 days at 10 mA cm^{-2}	This work

Table S2. Summary of the catalyst properties examined in .this work with other literatures. All performance were conducted at alkaline electrolyte of 1 M KOH. All reference sources are indicated in the manuscript.
